# Supplementary material for: AI-based detection and classification of anomalous aortic origin of coronary arteries using coronary CT angiography images
Source: Nat Commun. 2025 Apr 1;16:3095. doi: 10.1038/s41467-025-58362-9 (PMC11961624; doi:10.1038/s41467-025-58362-9)
Supplement: Supplementary file 1 — Supplementary Information [file 41467_2025_58362_MOESM1_ESM.pdf]

Supplementary Methods

The design and reporting of this study follows different dedicated guidelines for AI applications in medical imaging, including CLAIM (Checklist for Artificial Intelligence in Medical Imaging)[1], STARD-AI (Standards for Reporting of Diagnostic Accuracy Study-AI)[2], and MINIMAR (Minimum Information for Medical AI Reporting)[3]. Items of the above-listed guidelines documents have been jointly considered appropriate for the development, validation and testing of the AI-based model for automated AAOCA detection and classification in 3D-CCTA.

Evaluation of the models

Supplemental Figure 27 illustrates different strategies for model development and evaluation, which were implemented within our dataset using more labeled data in the model development More specific:

- Strategy 1: Model development was performed on the training dataset; the models were evaluated on the internal and external testing dataset with labeled cases. The external clinical testing dataset was used to evaluate the true and false positives, as the labeling was not available for this dataset.
- Strategy 2: Model training was performed on the entire dataset from Bern University Hospital. The labeled dataset from Zurich University Hospital served as an external testing dataset. The unlabeled open-access CCTA dataset (Guangdong Provincial People's Hospital) was used for external clinical evaluation, similar to Strategy 1.
- Strategy 3: Model training was performed on the entire datasets with labels, including data from Bern and Zurich University Hospitals. Following the previous strategy, external model performance was evaluated in the unlabeled dataset (external clinical evaluation dataset).

We report the results of Strategy 1 (Figure 2 in the main manuscript), while the results of Strategies 2 and 3 are reported in the **Supplementary material**. These additional strategies were explored to enhance the performance of the final model using the entire labeled dataset in different approaches. **Supplemental Figure 3** shows the different options for using the developed model in real clinical settings, from fully automated to semi-automated (physician in loop) approaches.

Model development

All data preprocessing and model development were conducted using different libraries in Python such as ITK[4,5], PyTorch[6], TorchIO[7], and MONAI[8] (more details are provided on GitHub)[9,10]. All computational was performed on high-performance servers equipped with 3 A100 GPUs, 250 CPU cores, and 1 TB of VRAM. All developed code and models are made publicly available on our AI-CVI laboratory's GitHub page (https://github.com/AI-in-Cardiovascular-Medicine/AAOCA). In addition, we have also provided a publicly available web service, accessible via the following link (Link to the project: https://mb-neuro.medical-blocks.ch/public_access/projects and link to the WebApp: https://mb-neuro.medical-blocks.ch/public_access/projects/aaoca), which allows users to easily upload images in various desired formats for use to get the report and result based on models developed in the current study.

Supplementary Discussion

Although we reported the whole results based on strategy one, our goal was to make it more generalizable and robust by utilizing all available labeled datasets for further use. The performance of the model remained consistent when tested with data from the external test set under the second strategy, and in both the second and third strategies, we did not find more positive cases from the external clinical evaluation dataset; however, the number of false positive cases decreased. This demonstrates the robustness and generalizability of the model developed using the first and main strategy. However, for future applications, we recommend developing the model using the third strategy, as it utilizes the entire dataset from multiple centers and is more likely to produce generalizable results in real-world scenarios.

Supplemental Figures

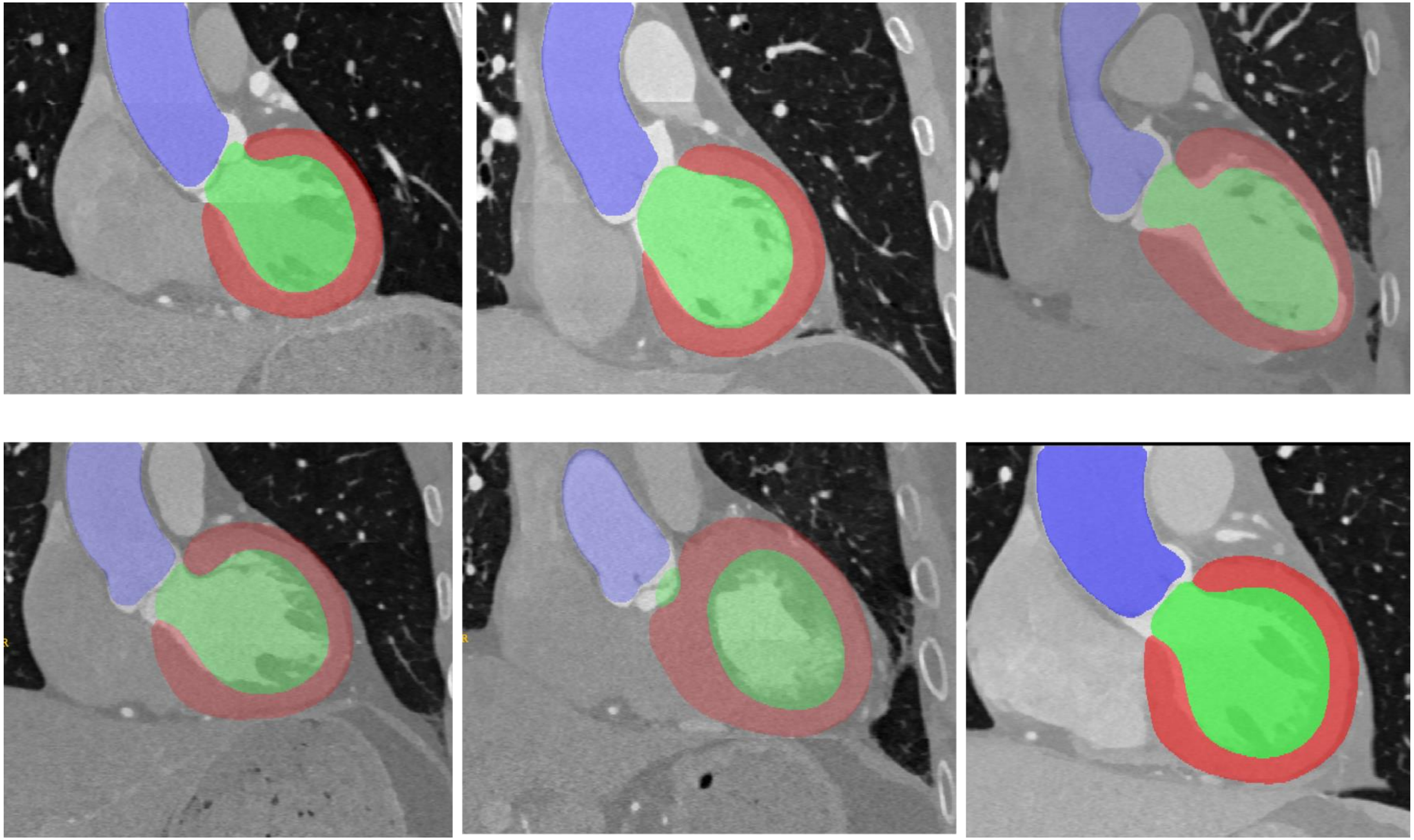

Supplemental Figure 1: Six different cases with their corresponding segmentations of the aorta and left ventricle.

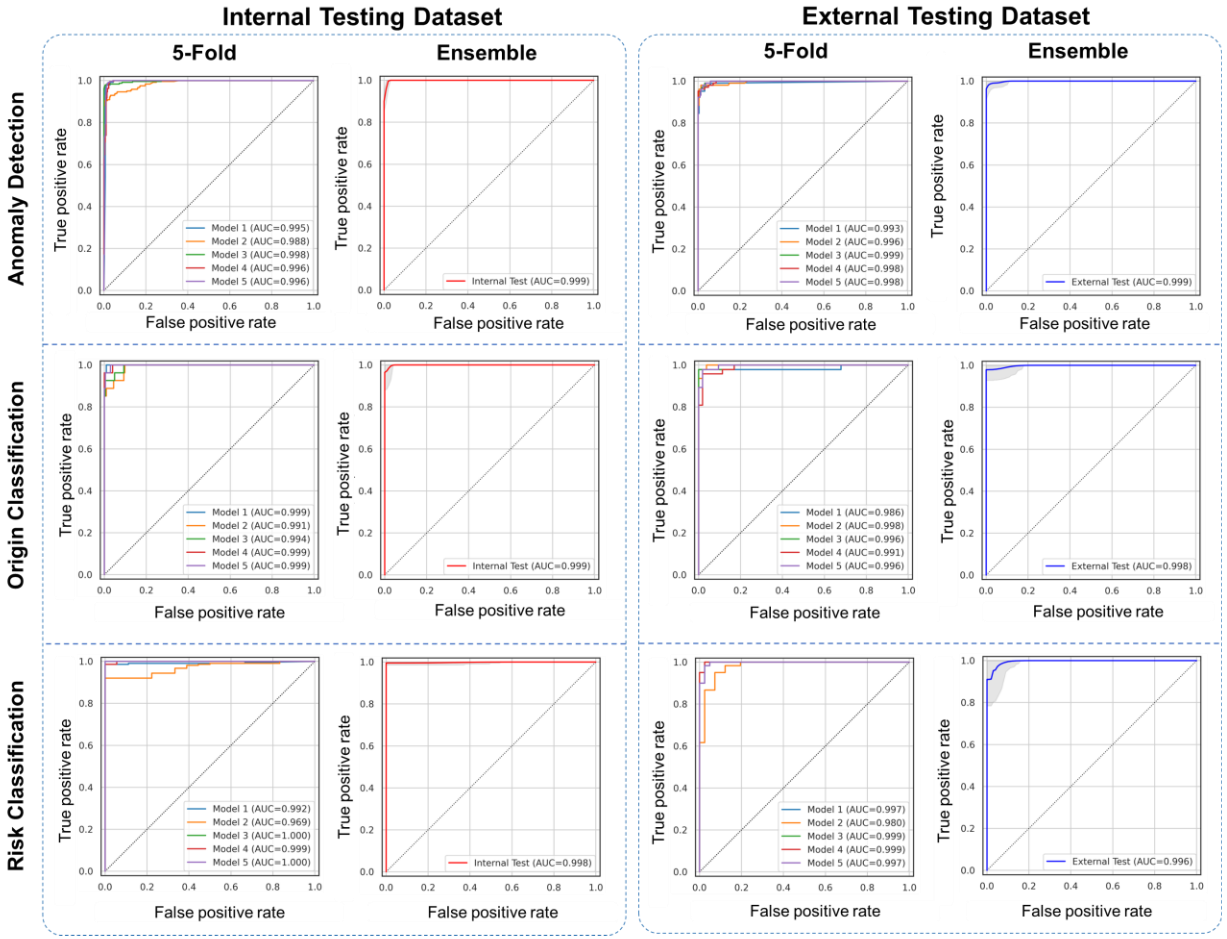

Supplemental Figure 2: ROC curves including the 5 different folds and the ensemble of 5 models across different tasks for various test datasets in the male population. Confidence intervals and tolerance intervals for the ensemble models were computed with the bootstrap method (10,000 iterations), the gray area on the ensemble figures is the tolerance interval. Anomaly Detection: distinguishing between normal cases and those with AAOCA; Origin Classification: classifying the anomalous vessel into either the right (R-AAOCA) or left (L-AAOCA); Risk Classification: scoring the AAOCA risk, classifying it as either low-risk or high-risk anatomy. AUC: area under the curve. Source data are provided as a Source Data file.

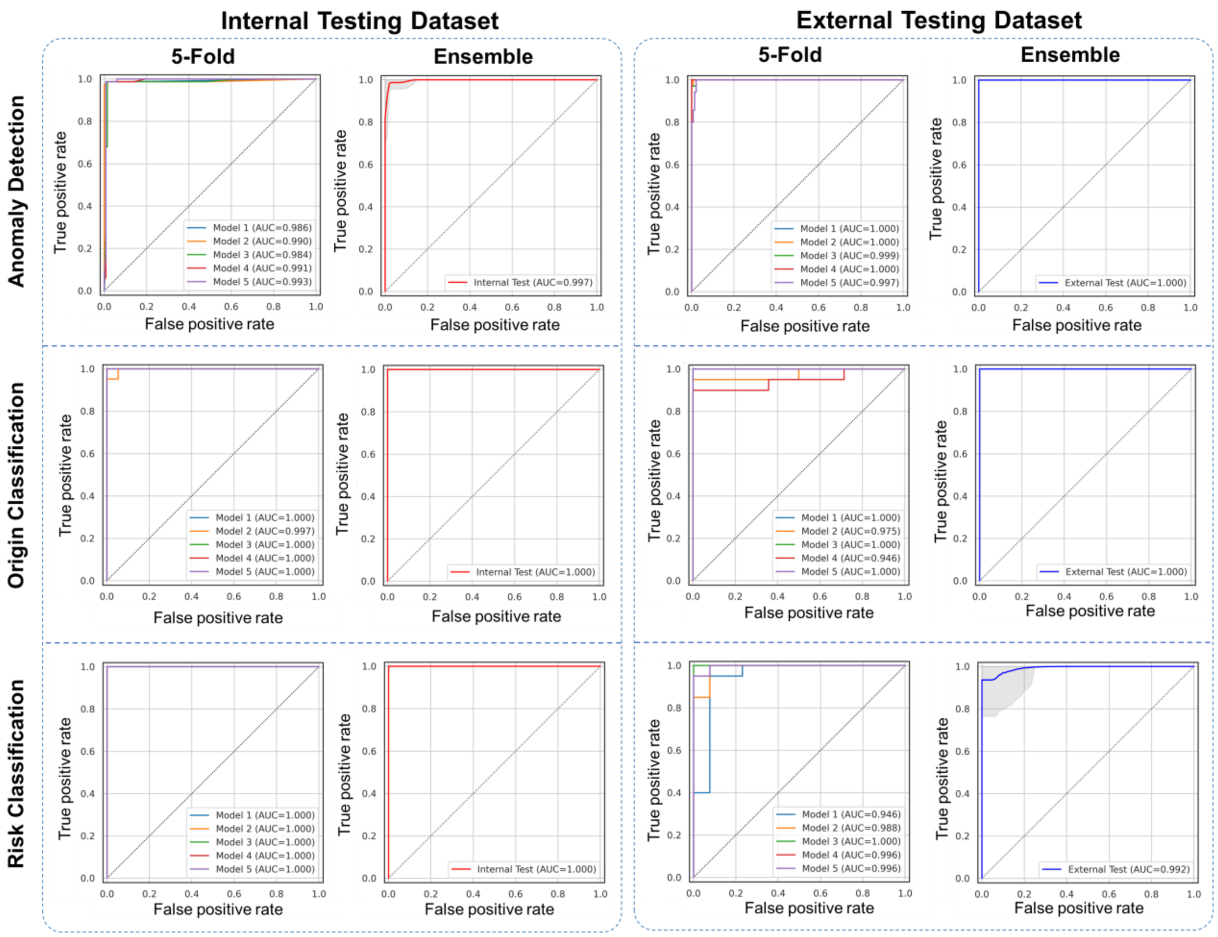

Supplemental Figure 3: ROC curves including the 5 different folds and the ensemble of 5 models across different tasks for various test datasets in the female population. Confidence intervals and tolerance intervals for the ensemble models were computed with the bootstrap method (10,000 iterations), the gray area on the ensemble figures is the tolerance interval. Anomaly Detection: distinguishing between normal cases and those with AAOCA; Origin Classification: classifying the anomalous vessel into either the right (R-AAOCA) or left (L-AAOCA); Risk Classification: scoring the AAOCA risk, classifying it as either low-risk or high-risk anatomy. AUC: area under the curve. Source data are provided as a Source Data file.

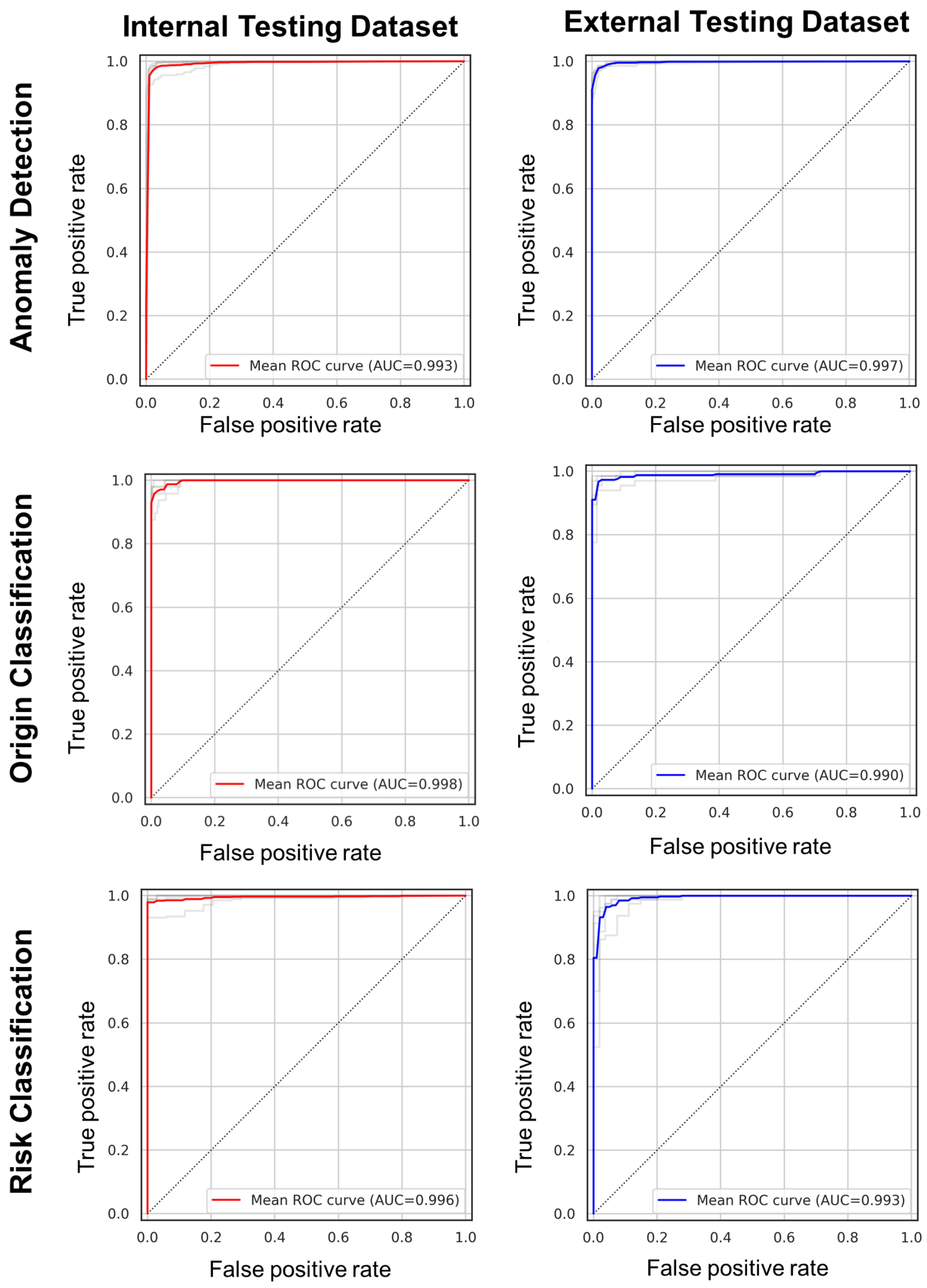

Supplemental Figure 4: Mean ROC curves of 5 different folds across different Tasks for various datasets. Anomaly Detection: distinguishing between normal cases and those with AAOCA; Origin Classification: classifying the anomalous vessel into either the right (R-AAOCA) or left (L-AAOCA; Risk Classification: classifying the AAOCA risk, classifying it as either low-risk or high-risk. Source data are provided as a Source Data file.

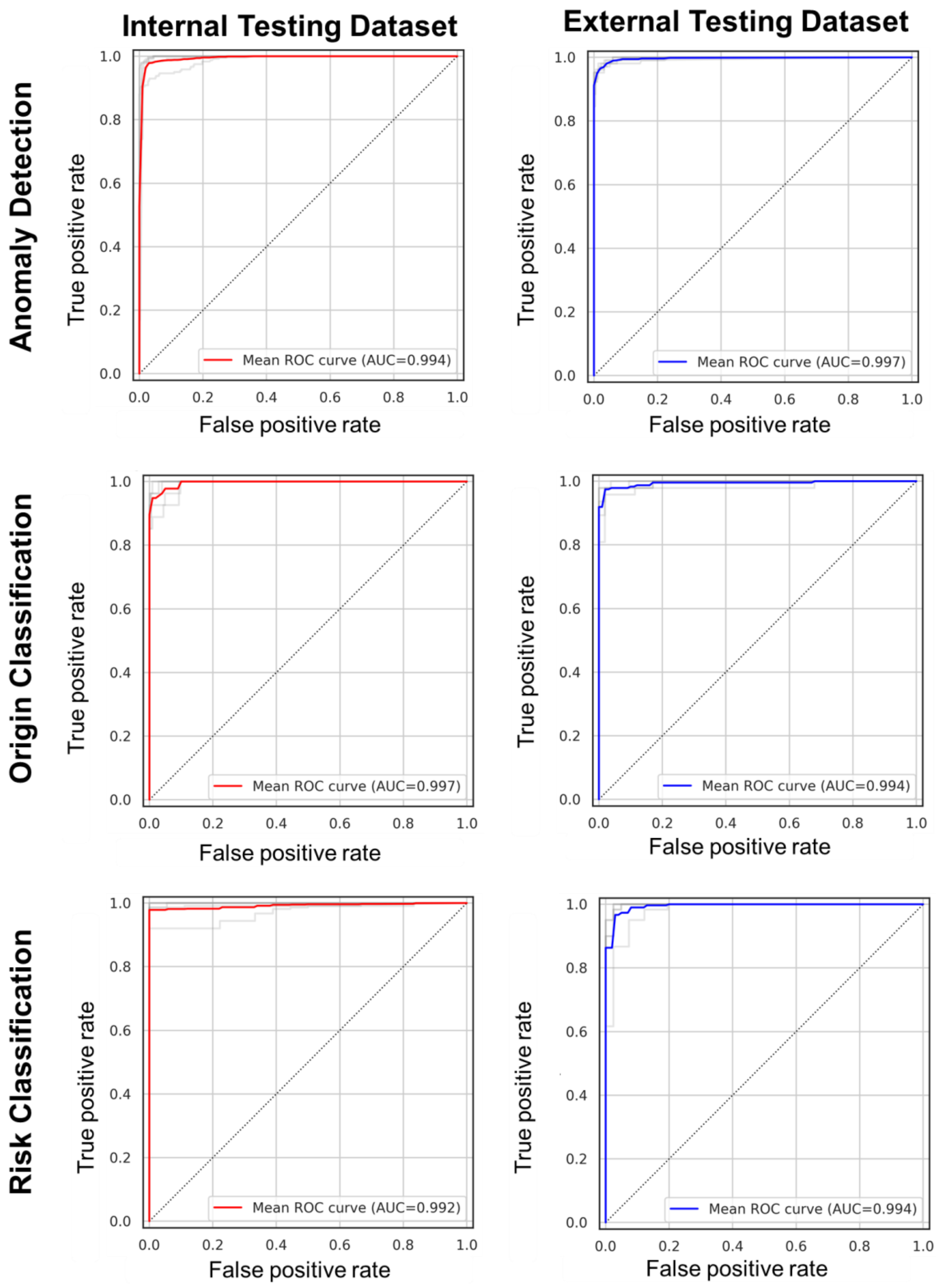

Supplemental Figure 5: Mean ROC curves of 5 different folds across different Tasks for various datasets for the male population. Anomaly Detection: distinguishing between normal cases and those with AAOCA; Origin Classification: classifying the anomalous vessel into either the right (R-AAOCA) or left (L-AAOCA; Risk Classification: classifying the AAOCA risk, classifying it as either low-risk or high-risk. Source data are provided as a Source Data file.

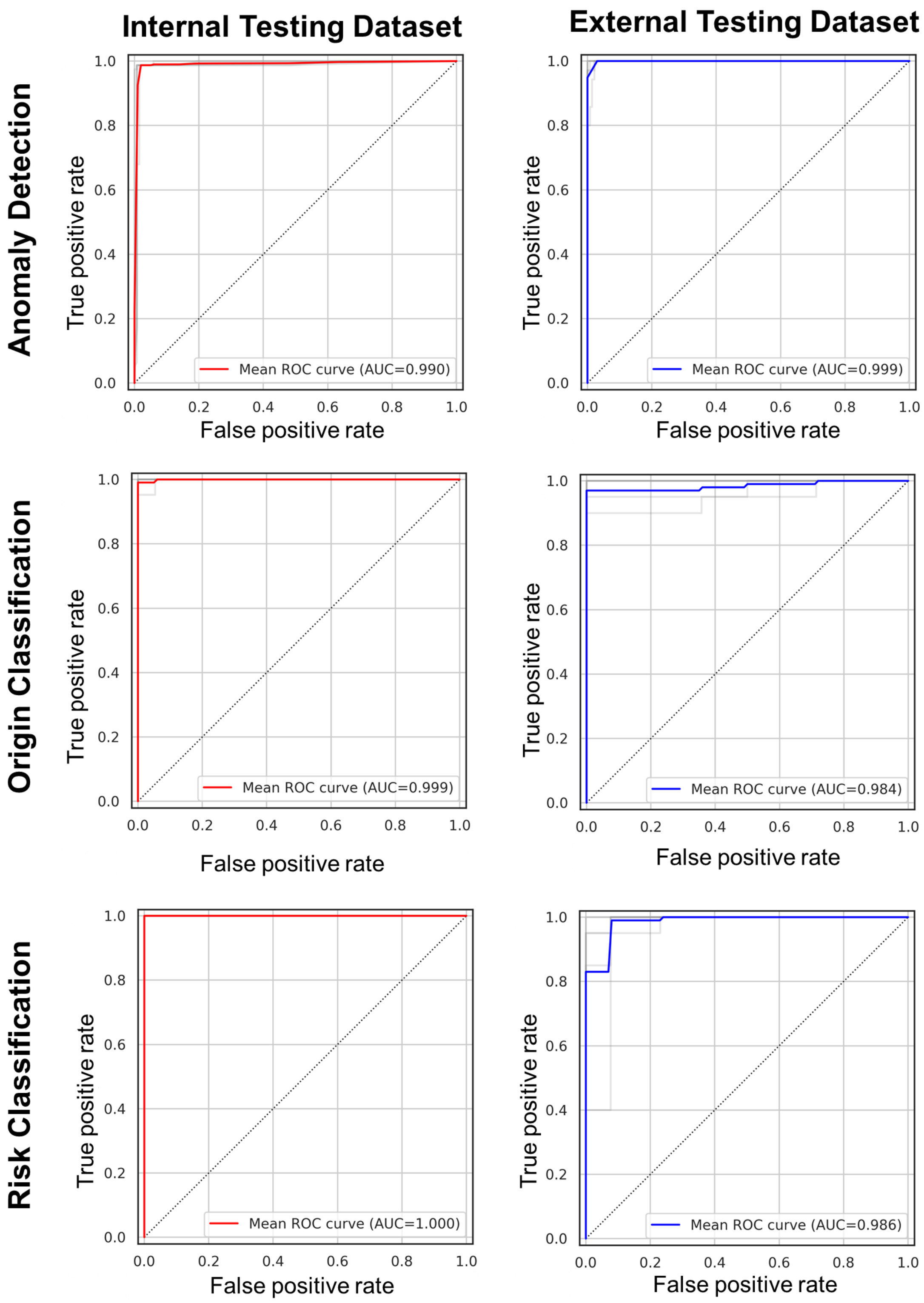

Supplemental Figure 6: Mean ROC curves of 5 different folds across different Tasks for various datasets for the female population. Anomaly Detection: distinguishing between normal cases and those with AAOCA; Origin Classification: classifying the anomalous vessel into either the right (R-AAOCA) or left (L-AAOCA; Risk Classification: classifying the AAOCA risk, classifying it as either low-risk or high-risk. Source data are provided as a Source Data file.

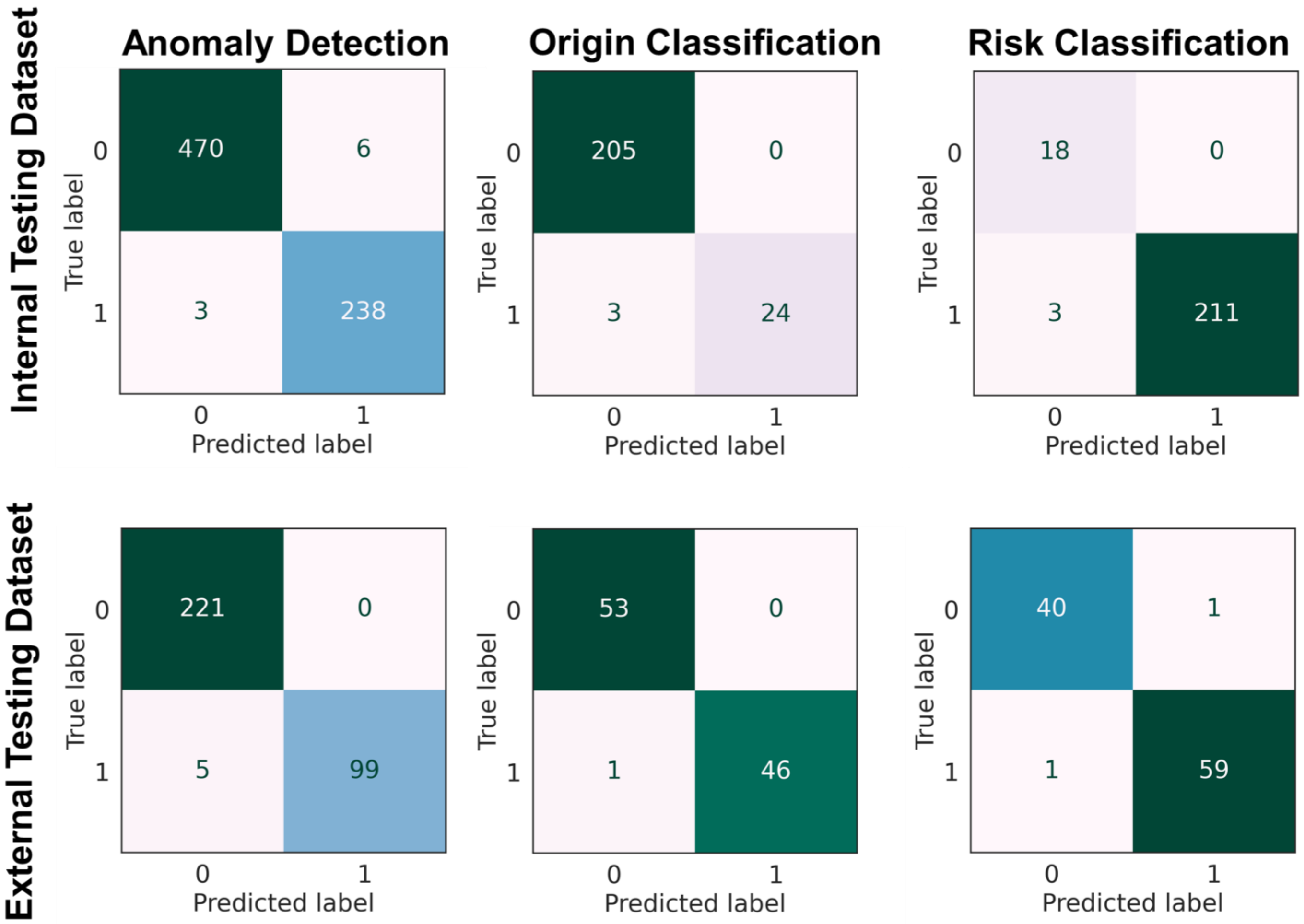

Supplemental Figure 7: Confusion matrices of different models in various Tasks for different datasets in the ensemble model for the male population. Anomaly Detection: distinguishing between normal cases and those with AAOCA; Origin Classification: classifying the anomalous vessel into either the right (R-AAOCA) or left (L-AAOCA); Risk Classification: scoring the AAOCA risk, classifying it as either low-risk or high-risk anatomy. Source data are provided as a Source Data file.

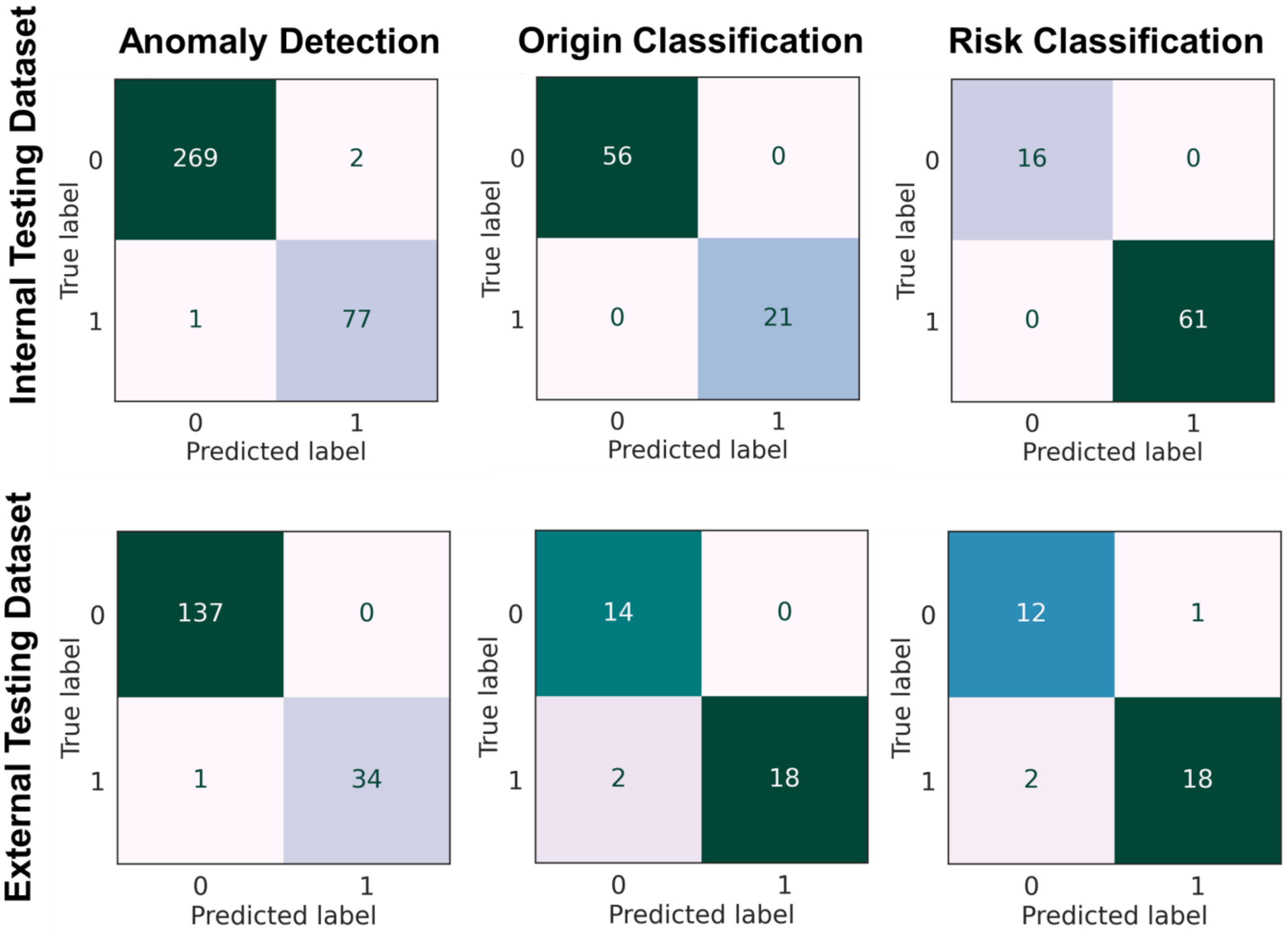

Supplemental Figure 8: Confusion matrices of different models in various Tasks for different datasets in the ensemble model for the female population. Anomaly Detection: distinguishing between normal cases and those with AAOCA; Origin Classification: classifying the anomalous vessel into either the right (R-AAOCA) or left (L-AAOCA); Risk Classification: scoring the AAOCA risk, classifying it as either low-risk or high-risk anatomy. Source data are provided as a Source Data file.

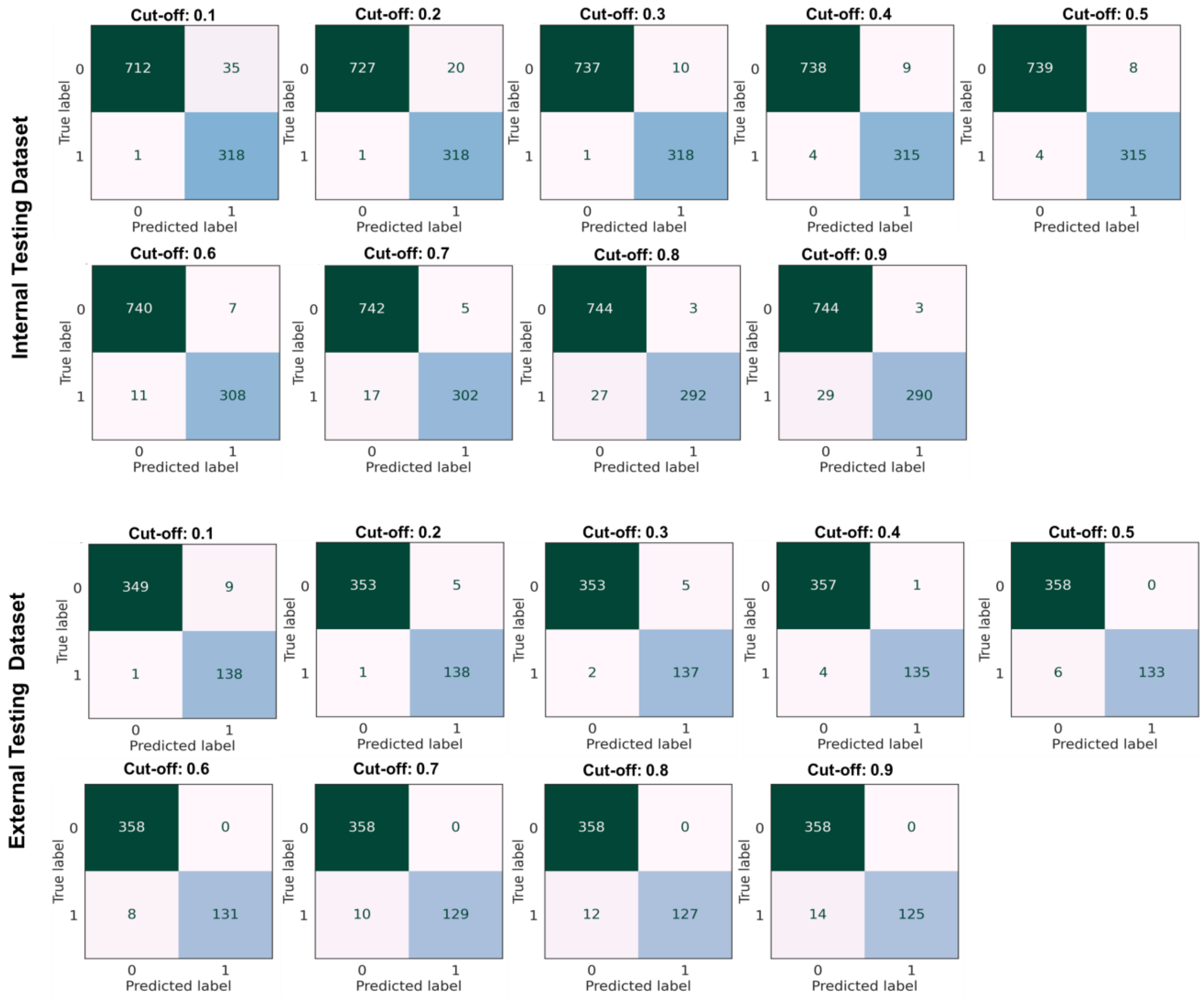

Supplemental Figure 9: Confusion matrices of ensemble model in Anomaly Detection for various datasets at different cut-off points. Anomaly Detection: distinguishing between normal cases and those with AAOCA. Source data are provided as a Source Data file.

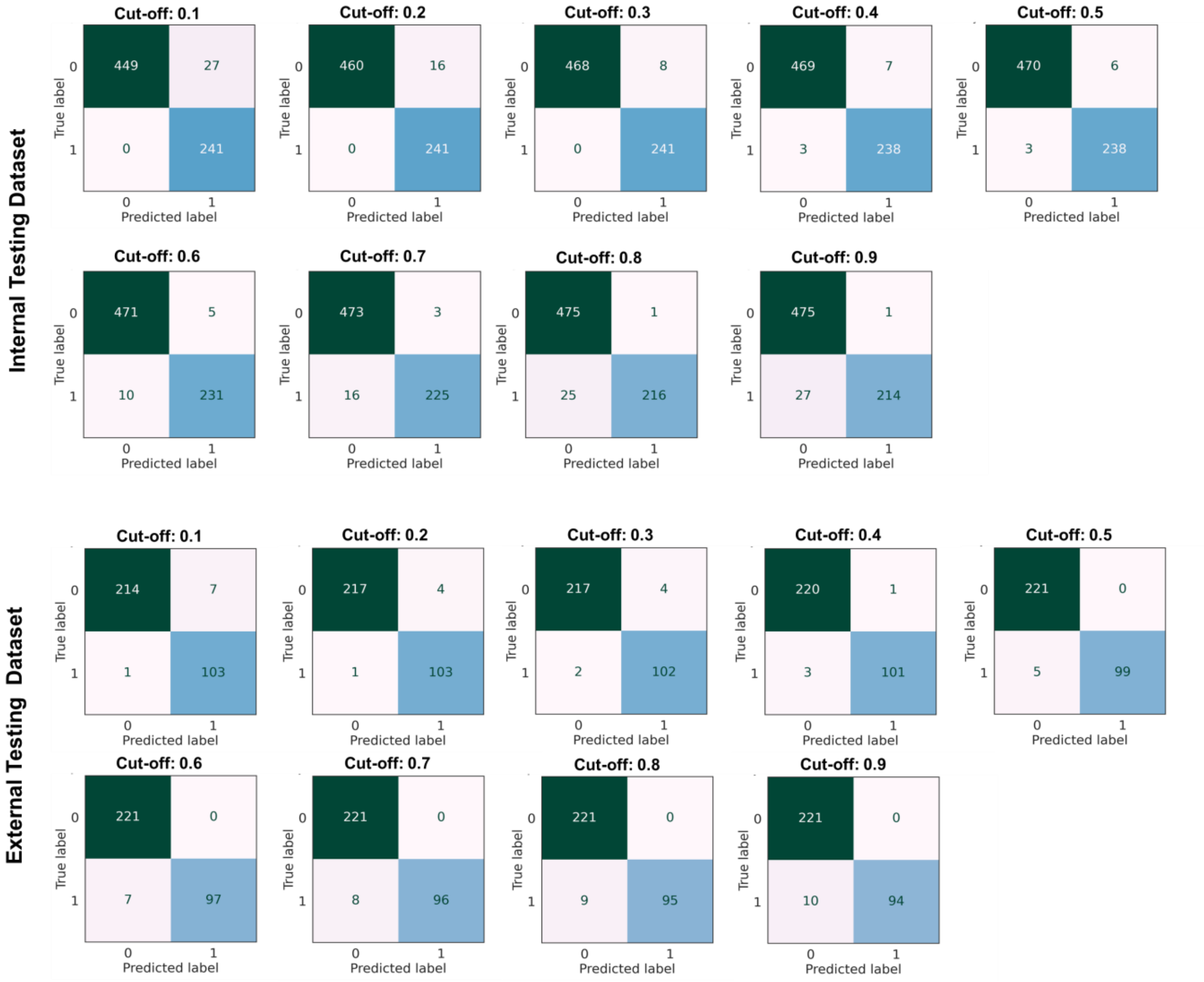

Supplemental Figure 10: Confusion matrices of ensemble model in Anomaly Detection for various datasets at different cut-off points in male population. Anomaly Detection: distinguishing between normal cases and those with AAOCA. Source data are provided as a Source Data file.

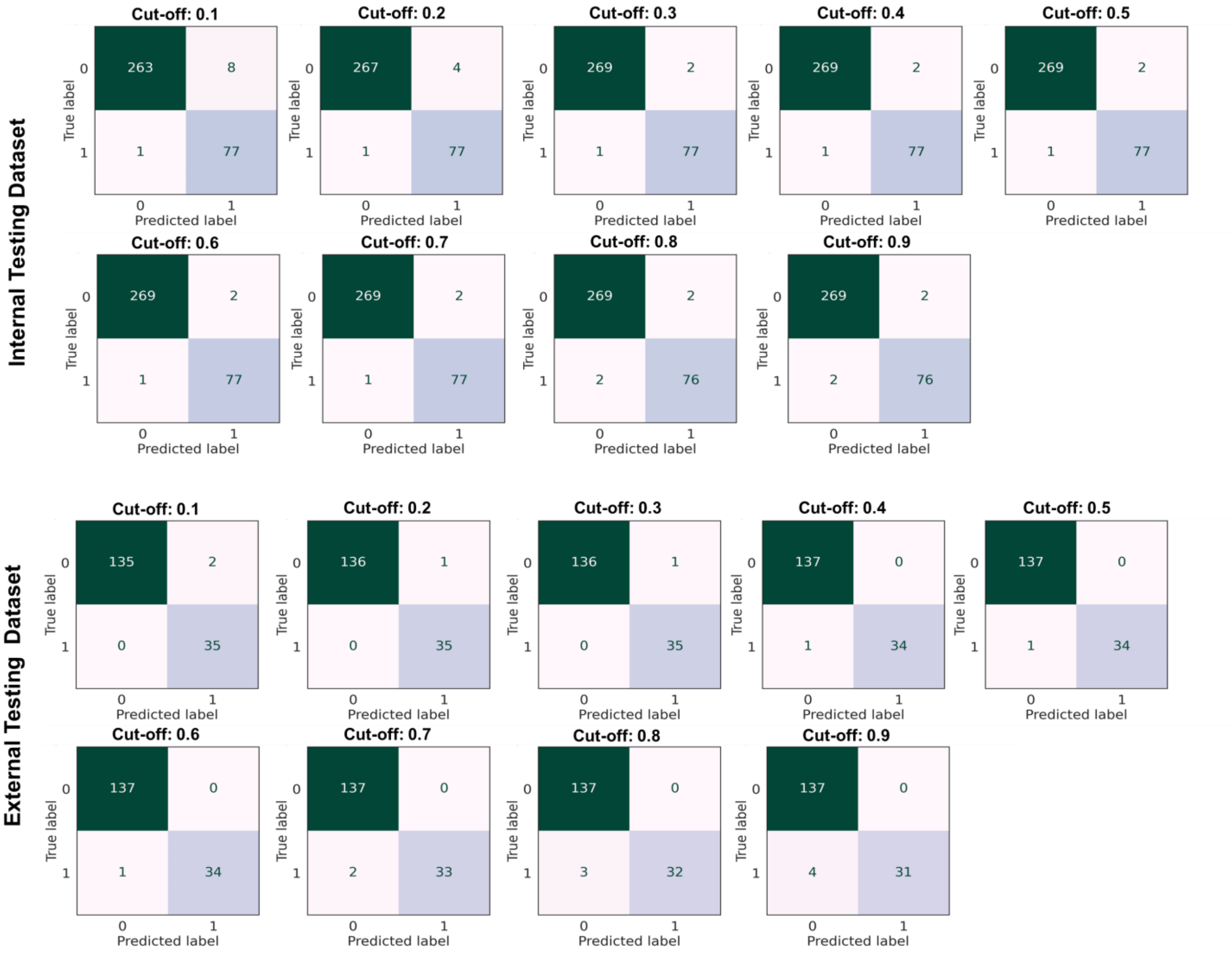

Supplemental Figure 11: Confusion matrices of ensemble model in Anomaly Detection for various datasets at different cut-off points in female population. Anomaly Detection: distinguishing between normal cases and those with AAOCA. Source data are provided as a Source Data file.

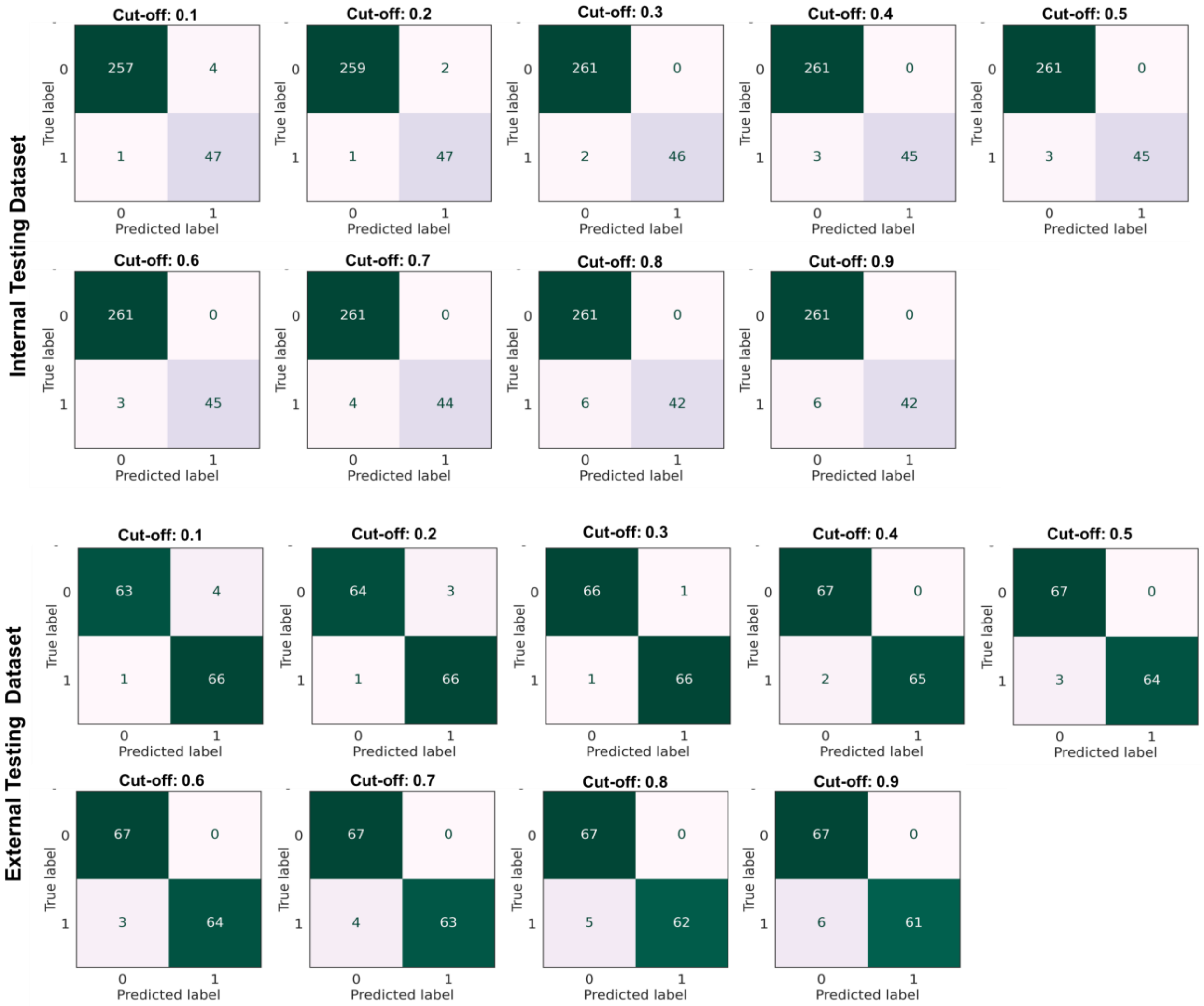

Supplemental Figure 12: Confusion matrices of ensemble model in Origin Classification for various datasets at different cut-off points. Origin Classification: classifying the anomalous vessel into either the right (R-AAOCA) or left (L-AAOCA). Source data are provided as a Source Data file.

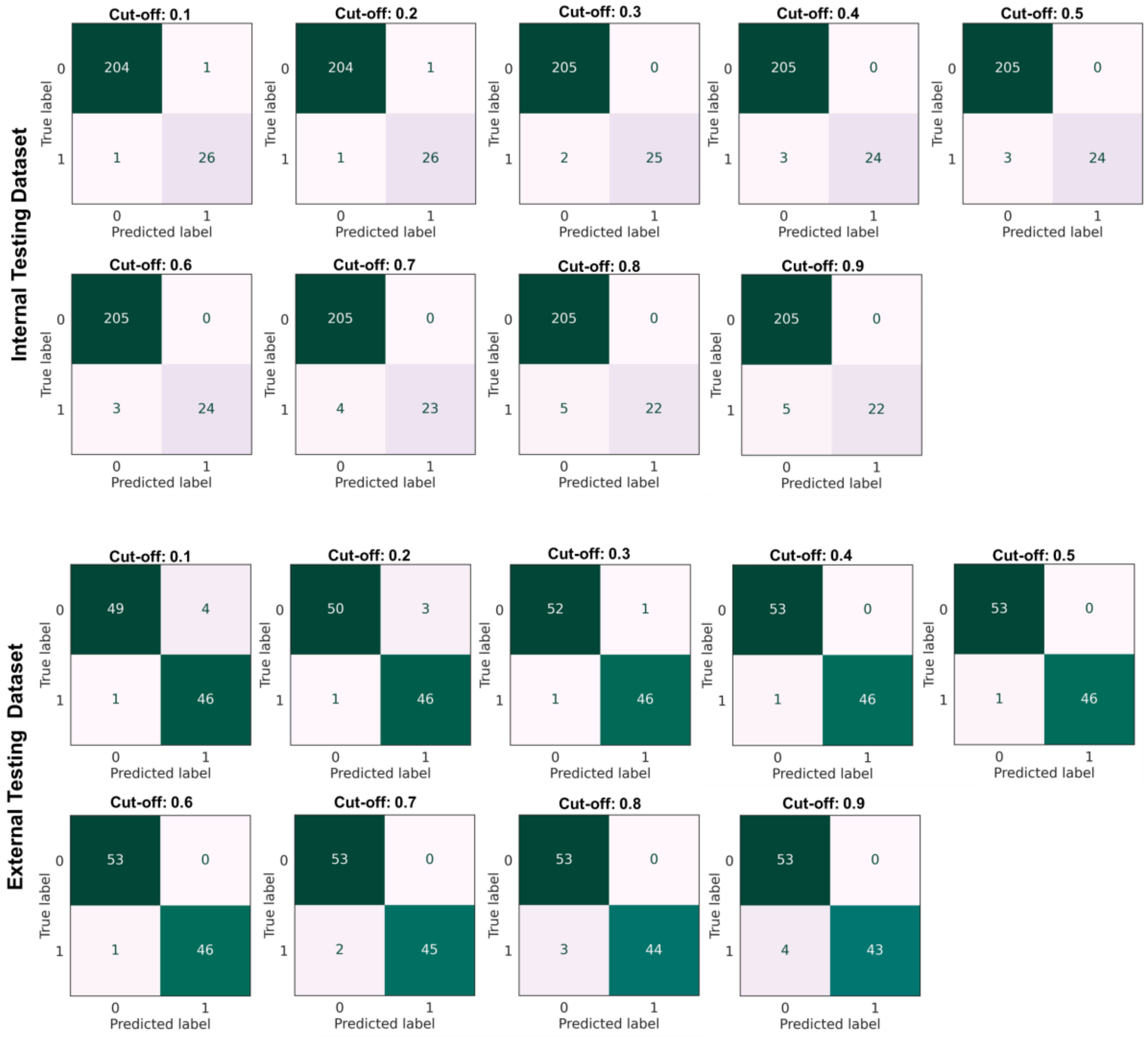

Supplemental Figure 13: Confusion matrices of ensemble model in Origin Classification for various datasets at different cut-off points in male population. Origin Classification: classifying the anomalous vessel into either the right (R-AAOCA) or left (L-AAOCA). Source data are provided as a Source Data file.

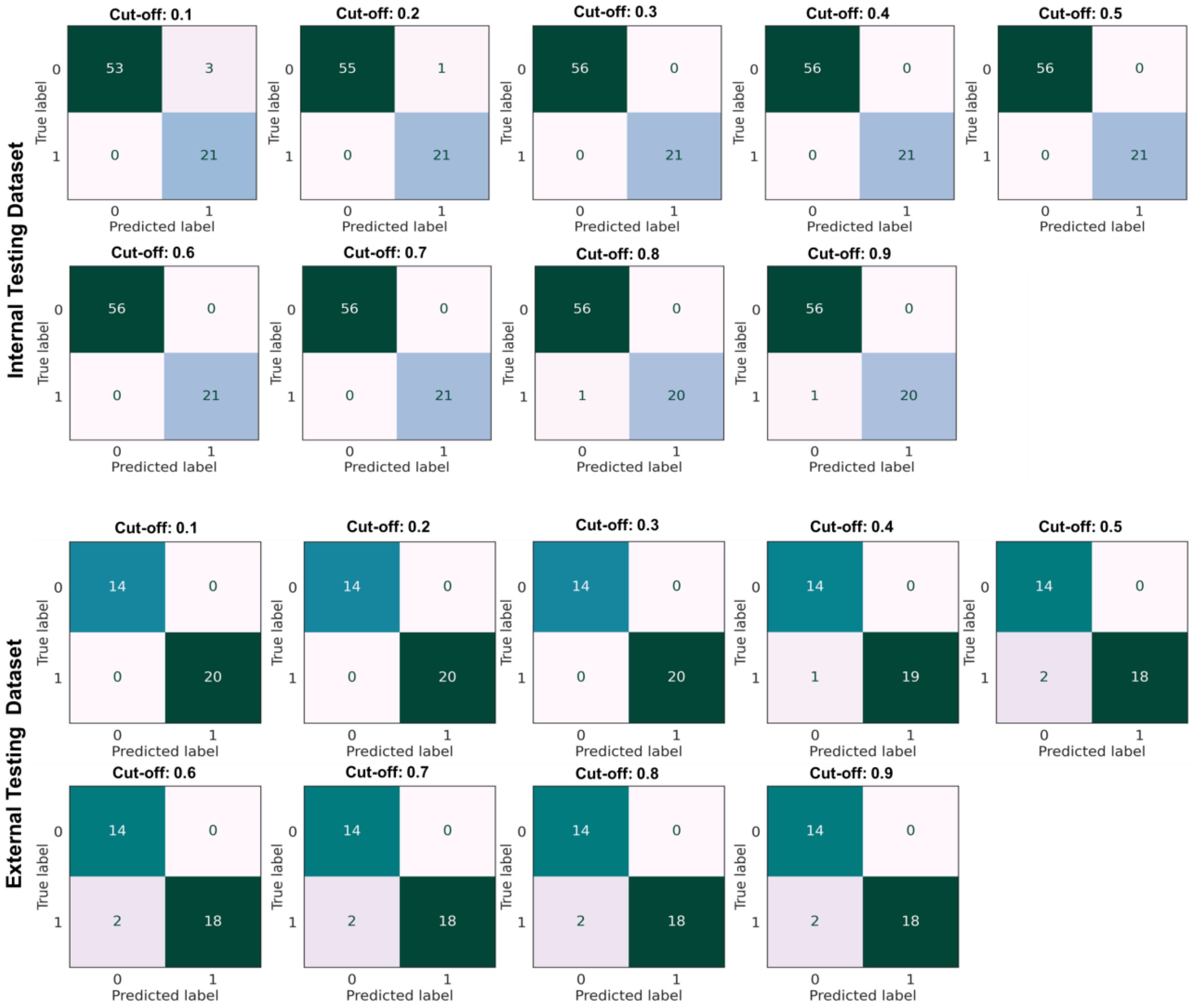

Supplemental Figure 14: Confusion matrices of ensemble model in Origin Classification for various datasets at different cut-off points in female population. Origin Classification: classifying the anomalous vessel into either the right (R-AAOCA) or left (L-AAOCA). Source data are provided as a Source Data file.

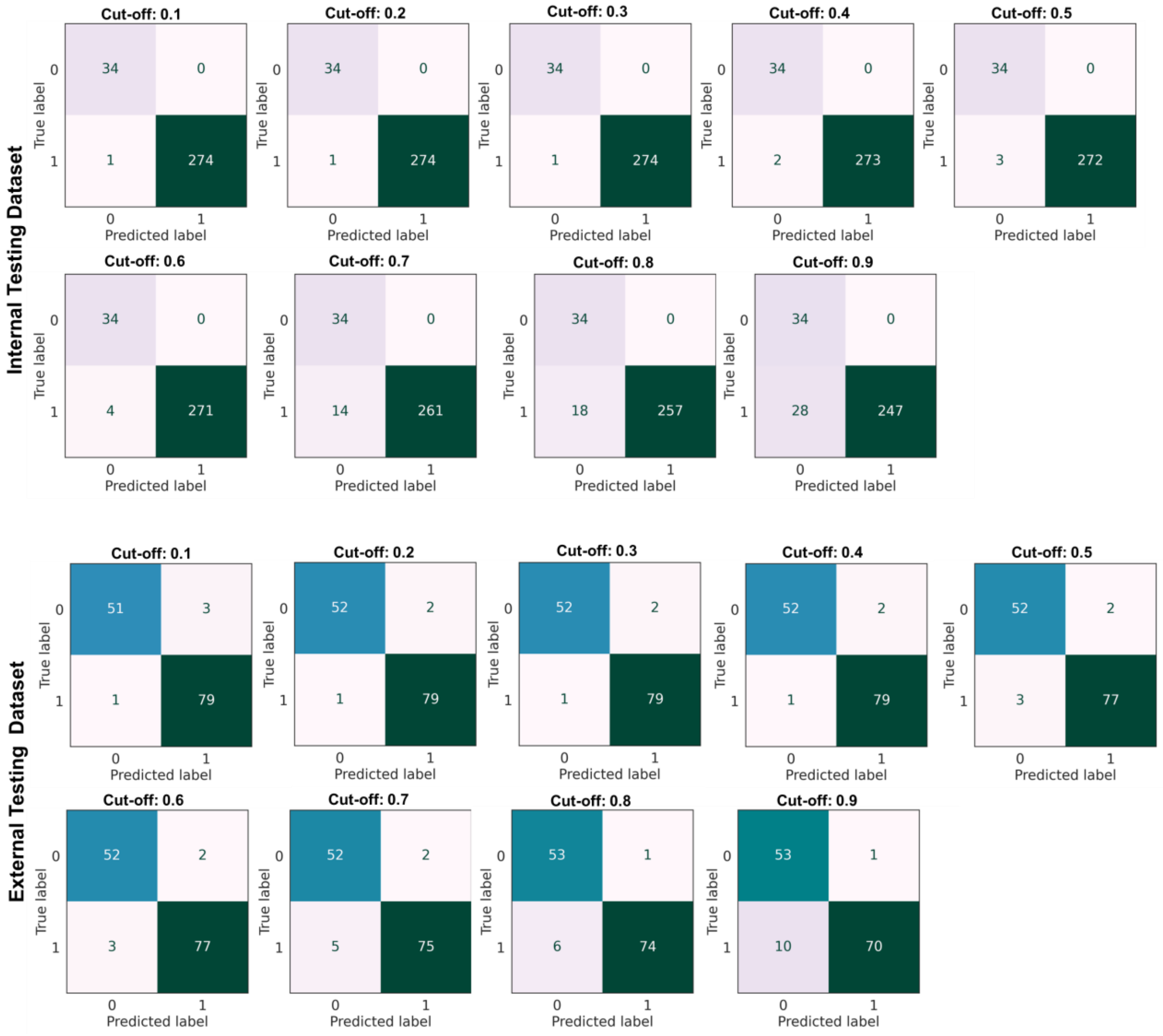

Supplemental Figure 15: Confusion matrices of ensemble model in Risk Classification for various datasets at different cut-off points. Risk Classification: classifying the AAOCA risk, classifying it as either low-risk or high-risk. Source data are provided as a Source Data file.

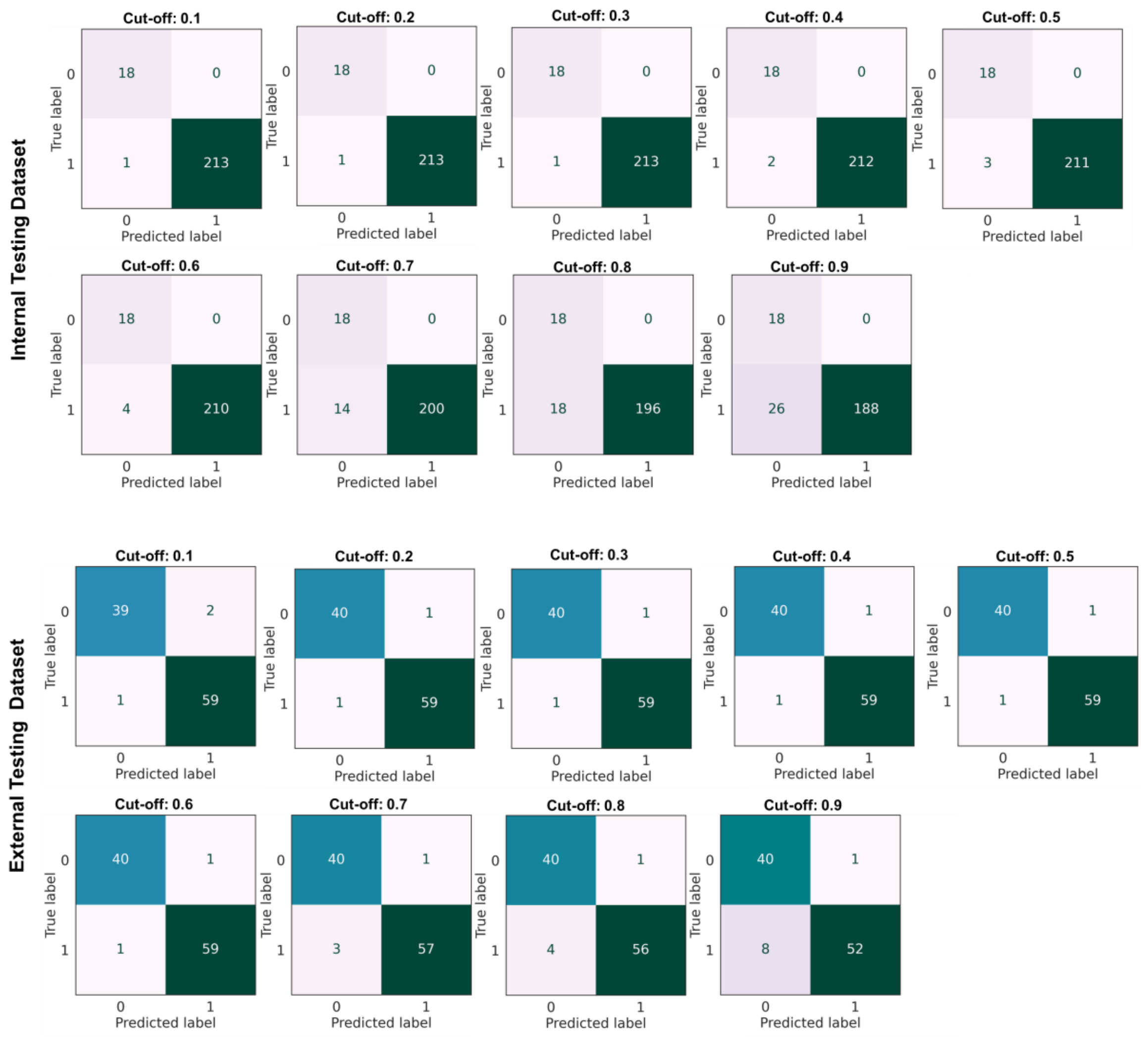

Supplemental Figure 16: Confusion matrices of ensemble model in Risk Classification for various datasets at different cut-off points in male population. Risk Classification: classifying the AAOCA risk, classifying it as either low-risk or high-risk. Source data are provided as a Source Data file.

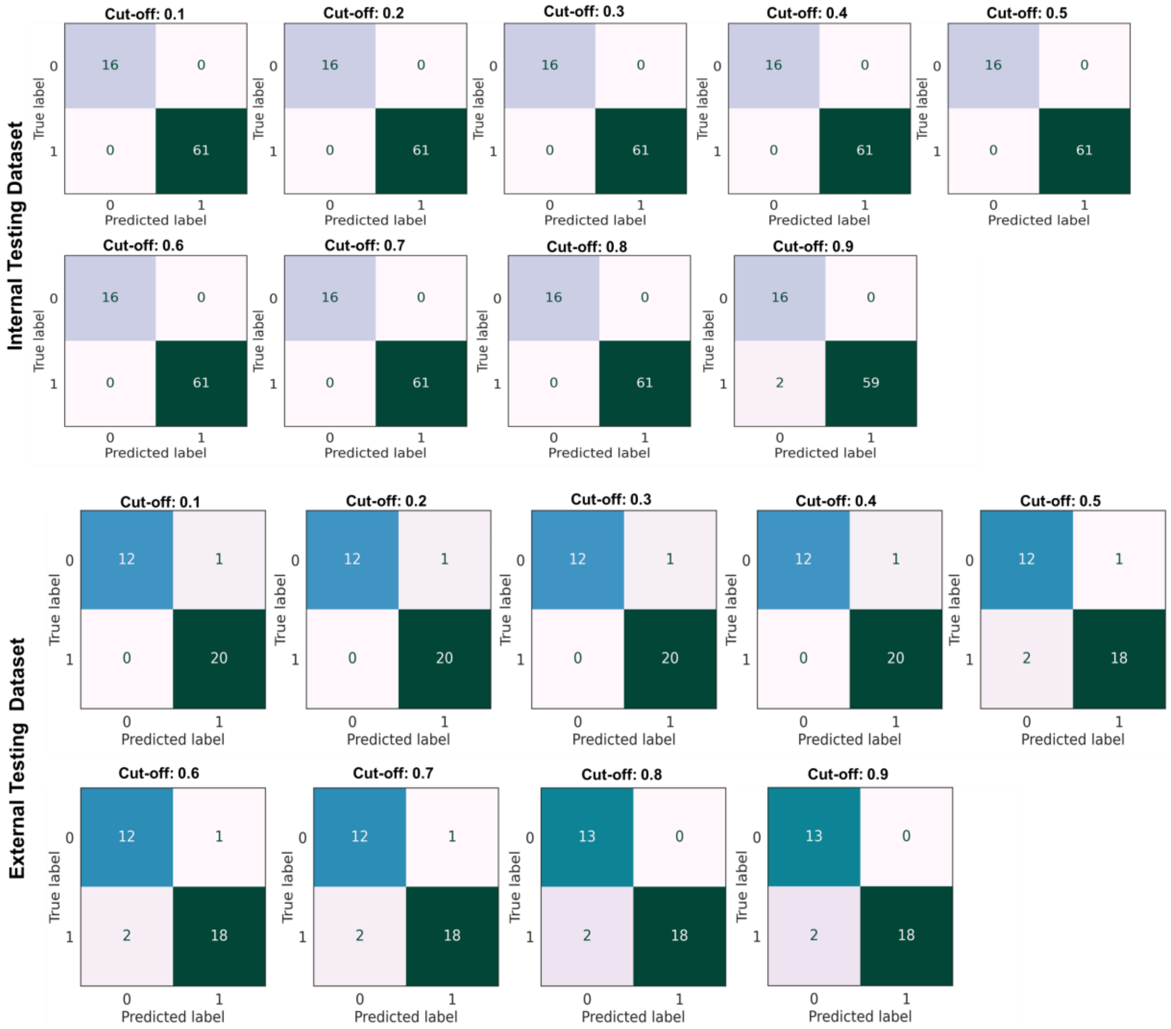

Supplemental Figure 17: Confusion matrices of ensemble model in Risk Classification for various datasets at different cut-off points in female population. Risk Classification: classifying the AAOCA risk, classifying it as either low-risk or high-risk. Source data are provided as a Source Data file.

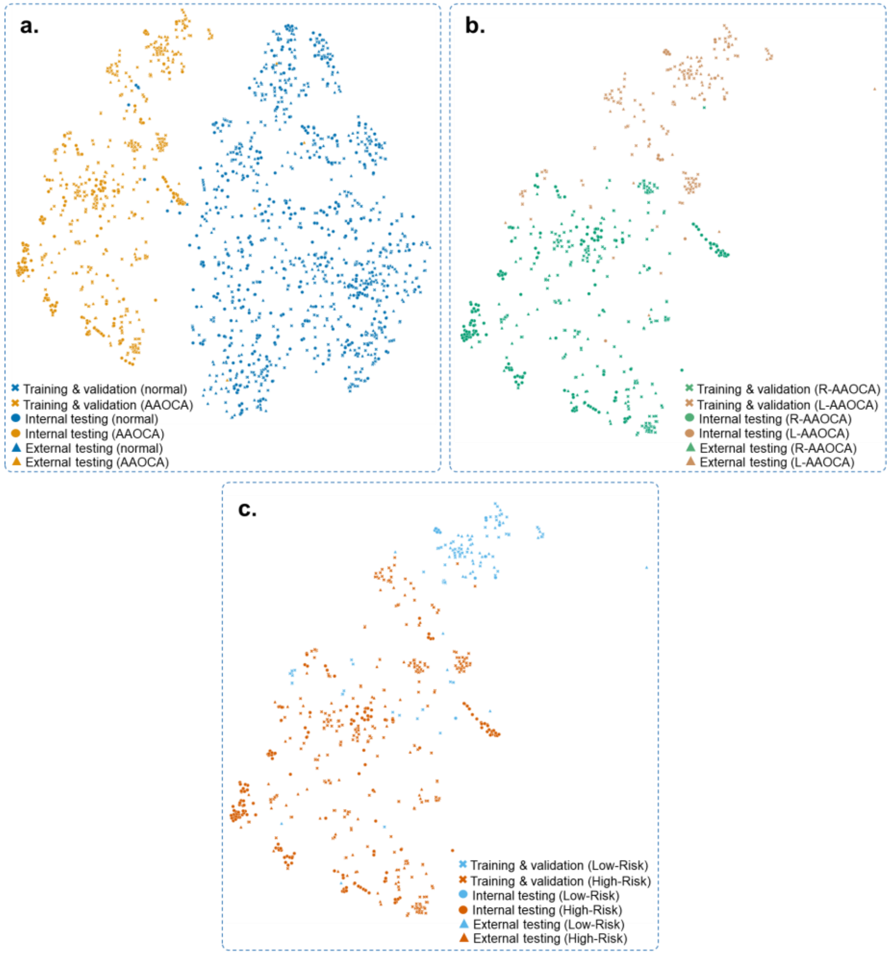

Supplemental Figure 18: t-SNE maps of the Anomaly Detection model in male population colorized for **a)** anomalies and normal cases. Only for the anomaly dataset, **b)** right and left anomalies, and **c)** high and low-risk anomalies. t-SNE: t-distributed stochastic neighbor embedding, AAOCA: Anomalous Aortic Origin of the Coronary Artery, CCTA: Coronary CT Angiography, R-AAOCA: Right AAOCA, L-AAOCA: left AAOCA. Source data are provided as a Source Data file.

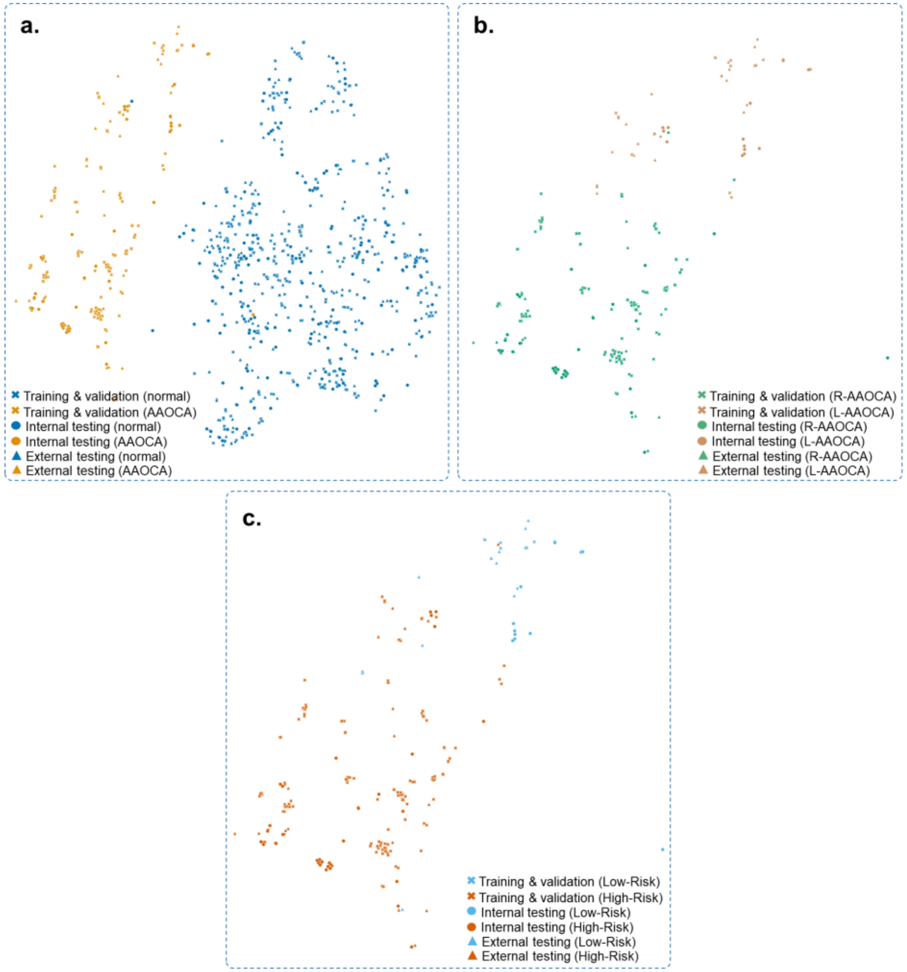

Supplemental Figure 19: t-SNE maps of the Anomaly Detection model in female population colorized for **a)** anomalies and normal cases. Only for the anomaly dataset, **b)** right and left anomalies, and **c)** high and low-risk anomalies. t-SNE: t-distributed stochastic neighbor embedding, AAOCA: Anomalous Aortic Origin of the Coronary Artery, CCTA: Coronary CT Angiography, R-AAOCA: Right AAOCA, L-AAOCA: left AAOCA. Source data are provided as a Source Data file.

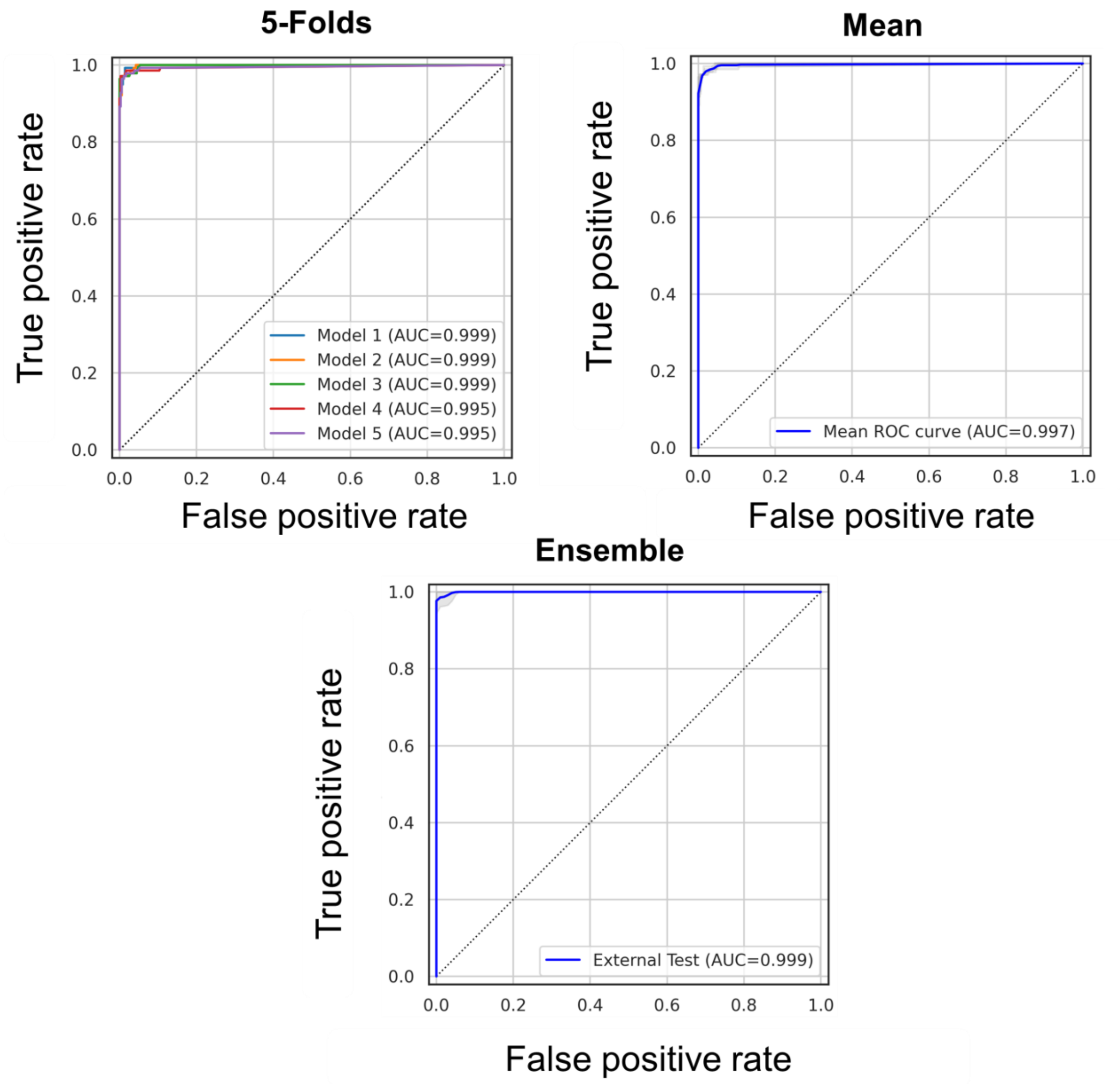

Supplemental Figure 20: ROC curves of 5 folds, mean, and an ensemble of strategy 2 in the external test dataset from external testing dataset. Anomaly Detection: distinguishing between normal cases and those with AAOCA. Source data are provided as a Source Data file. Confidence intervals and tolerance intervals for the ensemble models were computed with the bootstrap method (10,000 iterations), the gray area on the ensemble figures is the tolerance interval

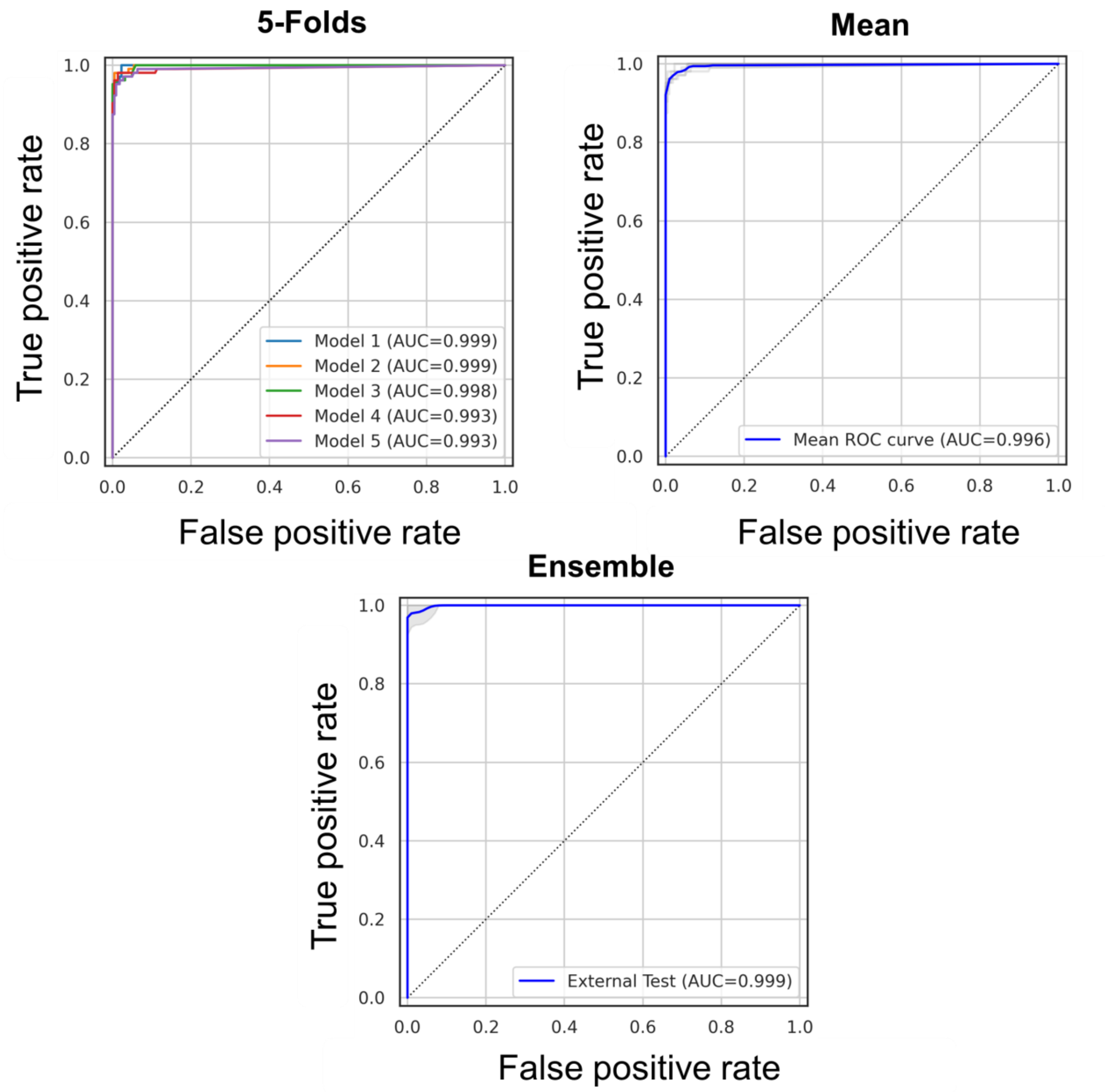

Supplemental Figure 21: ROC curves of 5 folds, mean, and an ensemble of strategy 2 in the external test dataset from external testing dataset for the male population. Anomaly Detection: distinguishing between normal cases and those with AAOCA. Source data are provided as a Source Data file. Confidence intervals and tolerance intervals for the ensemble models were computed with the bootstrap method (10,000 iterations), the gray area on the ensemble figures is the tolerance interval

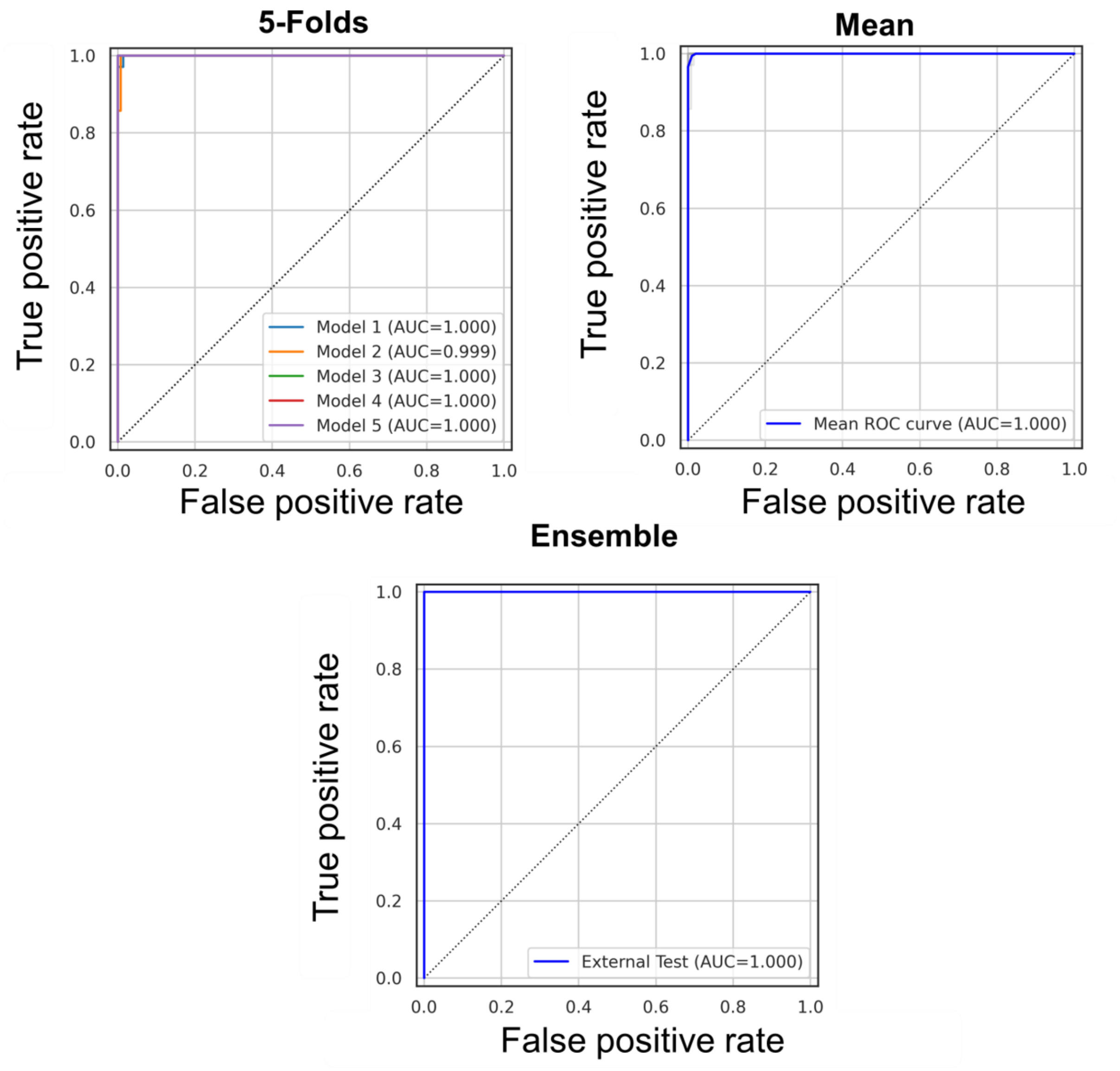

Supplemental Figure 22: ROC curves of 5 folds, mean, and an ensemble of strategy 2 in the external test dataset from external testing dataset for the female population. Anomaly Detection: distinguishing between normal cases and those with AAOCA. Source data are provided as a Source Data file. Confidence intervals and tolerance intervals for the ensemble models were computed with the bootstrap method (10,000 iterations), the gray area on the ensemble figures is the tolerance interval

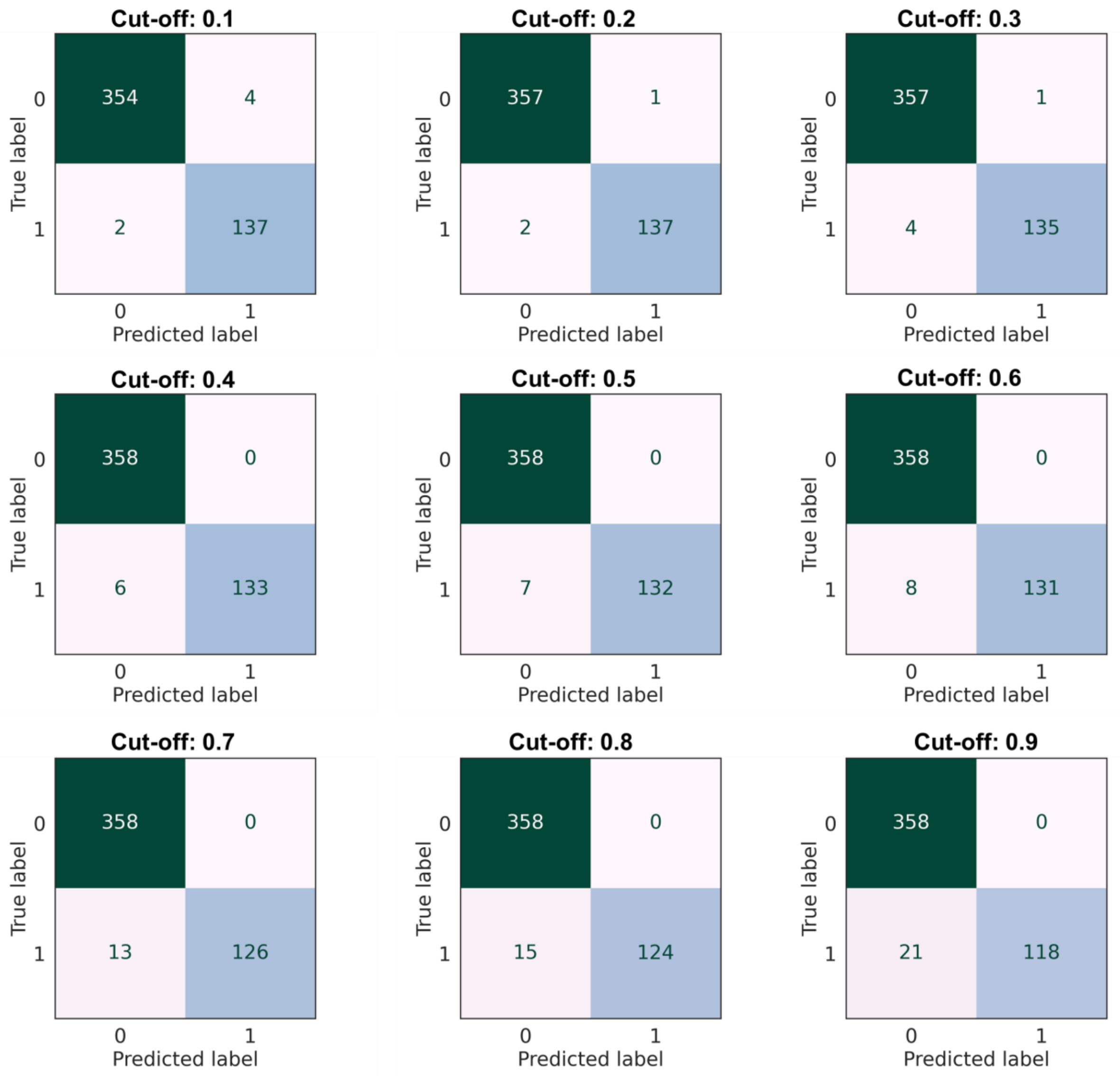

Supplemental Figure 23: Confusion matrices of ensemble model in Anomaly Detection in strategy 2 for external testing datasetat different cut-off points. Anomaly Detection: distinguishing between normal cases and those with AAOCA. Source data are provided as a Source Data file.

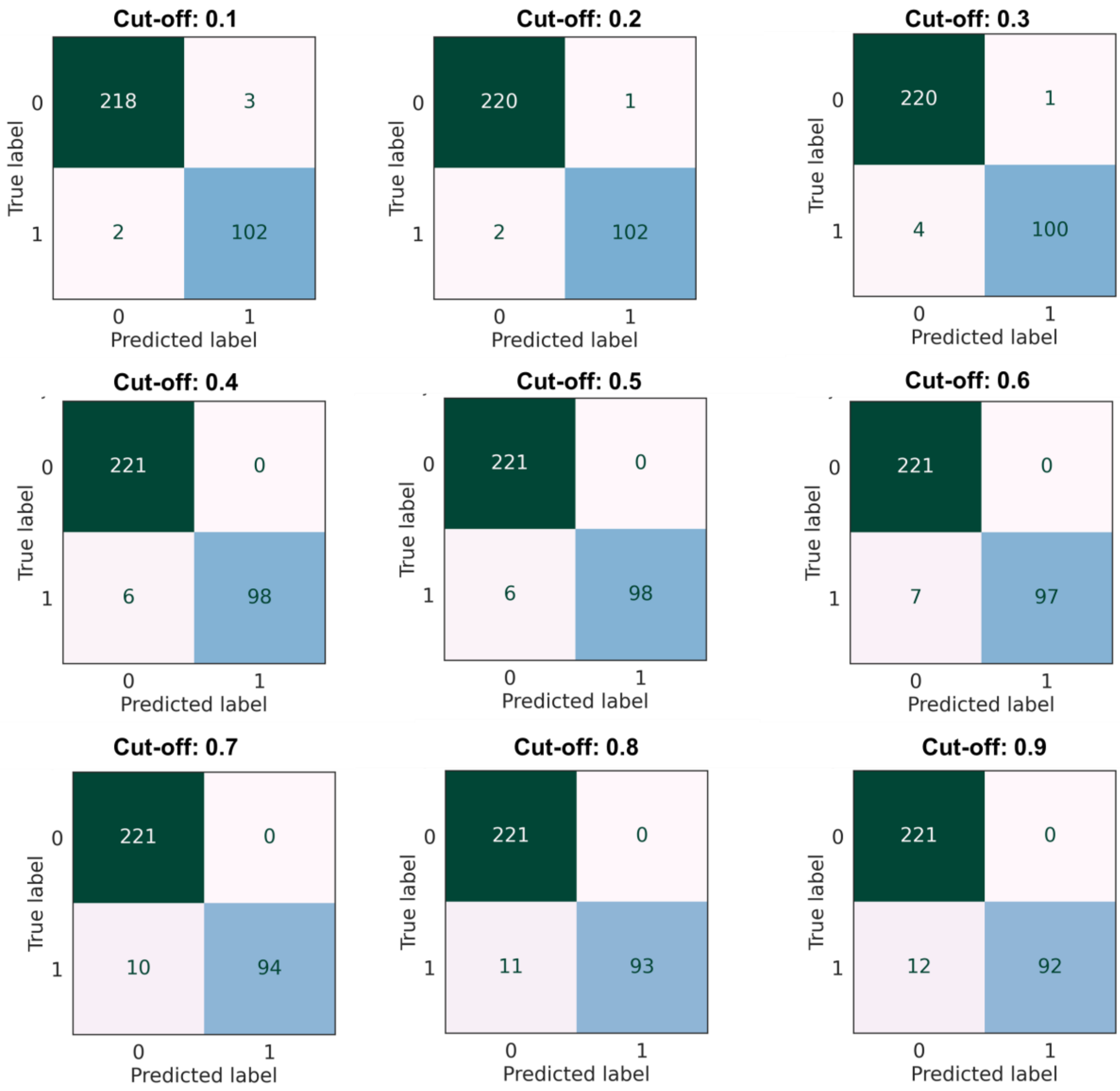

Supplemental Figure 24: Confusion matrices of ensemble model in Anomaly Detection in strategy 2 for external testing dataset at different cut-off points for the male population. Anomaly Detection: distinguishing between normal cases and those with AAOCA. Source data are provided as a Source Data file.

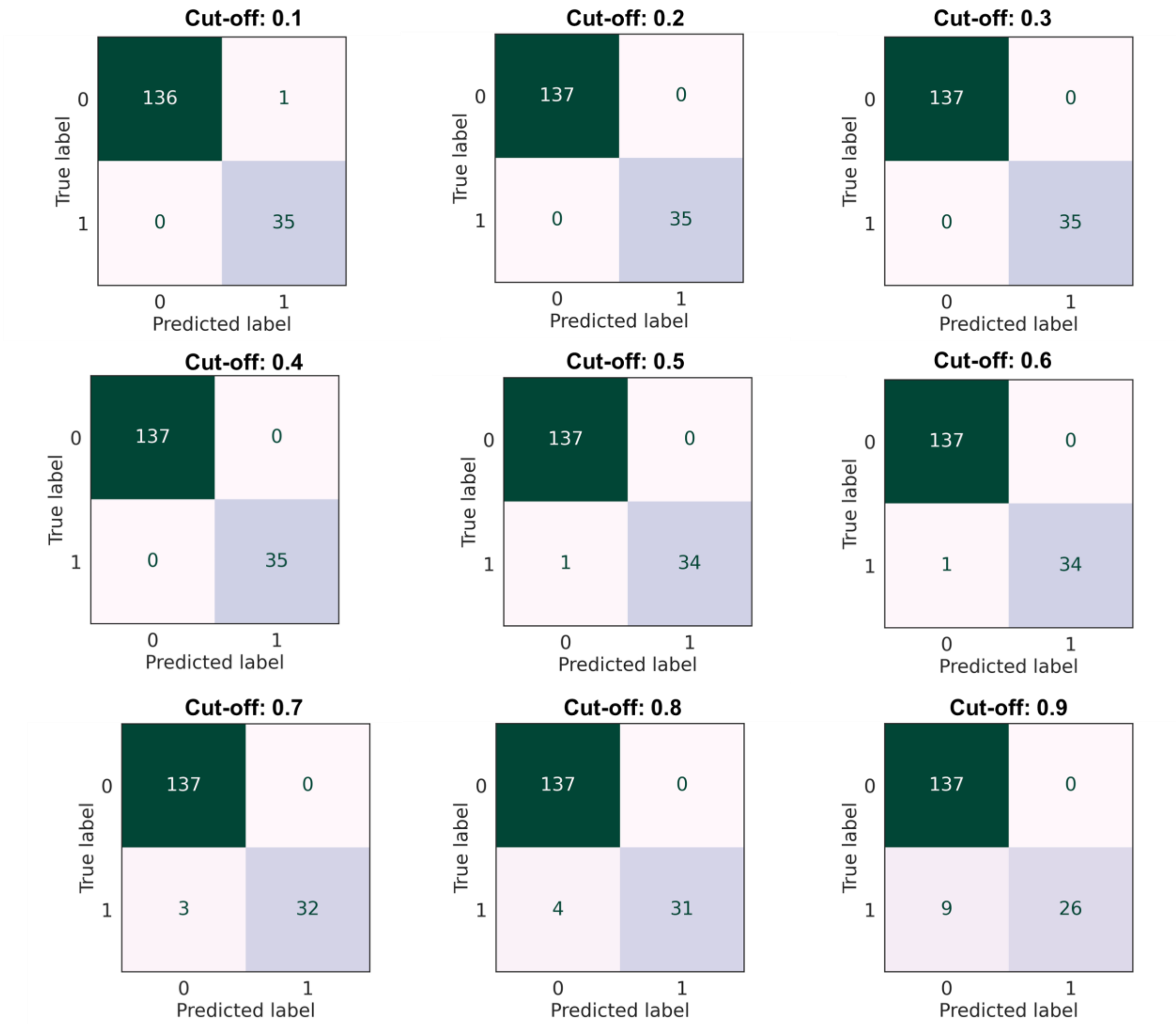

Supplemental Figure 25: Confusion matrices of ensemble model in Anomaly Detection in strategy 2 for external testing dataset at different cut-off points for the female population. Anomaly Detection: distinguishing between normal cases and those with AAOCA. Source data are provided as a Source Data file.

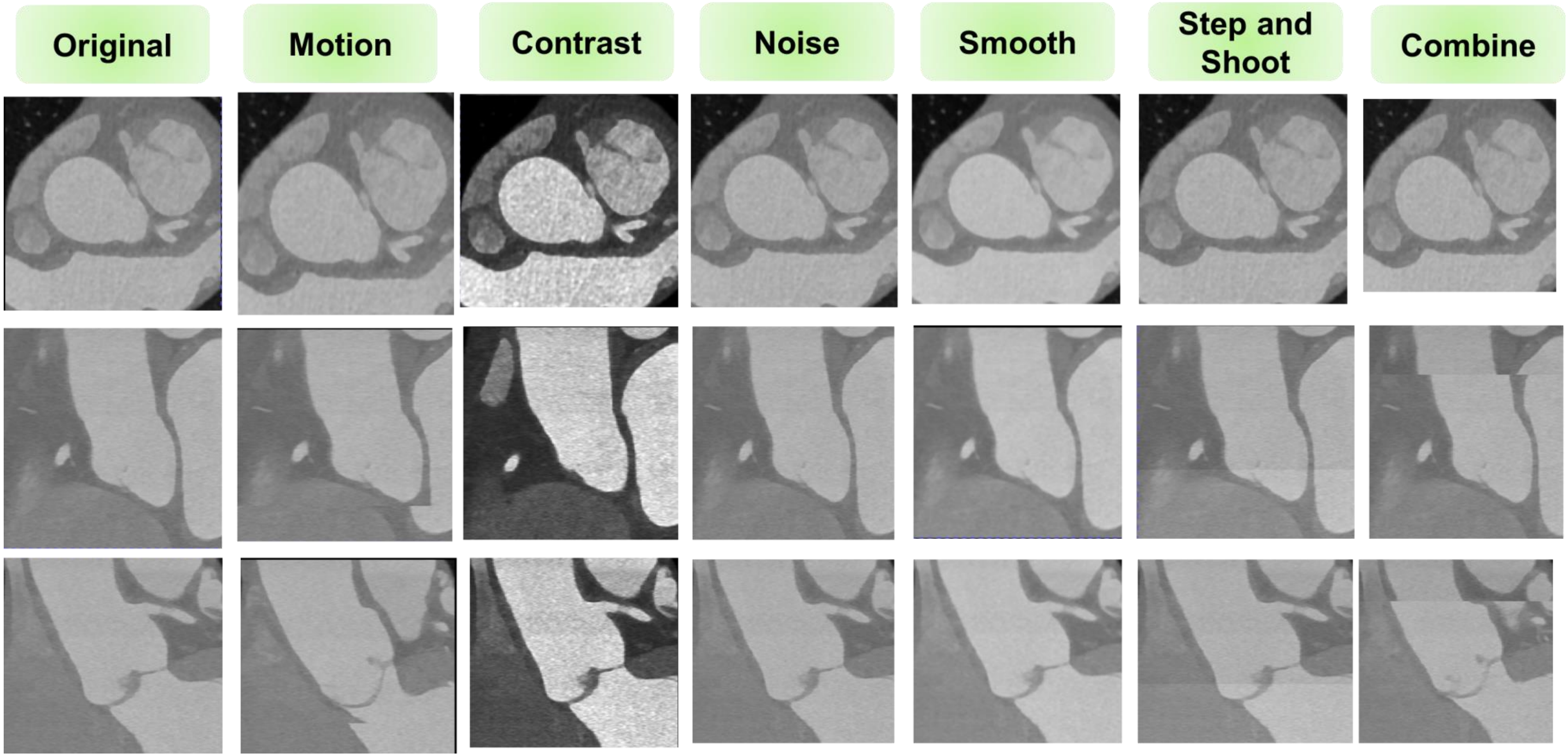

Supplemental Figure 26: Original image and different augmentations applied to the image in different views.

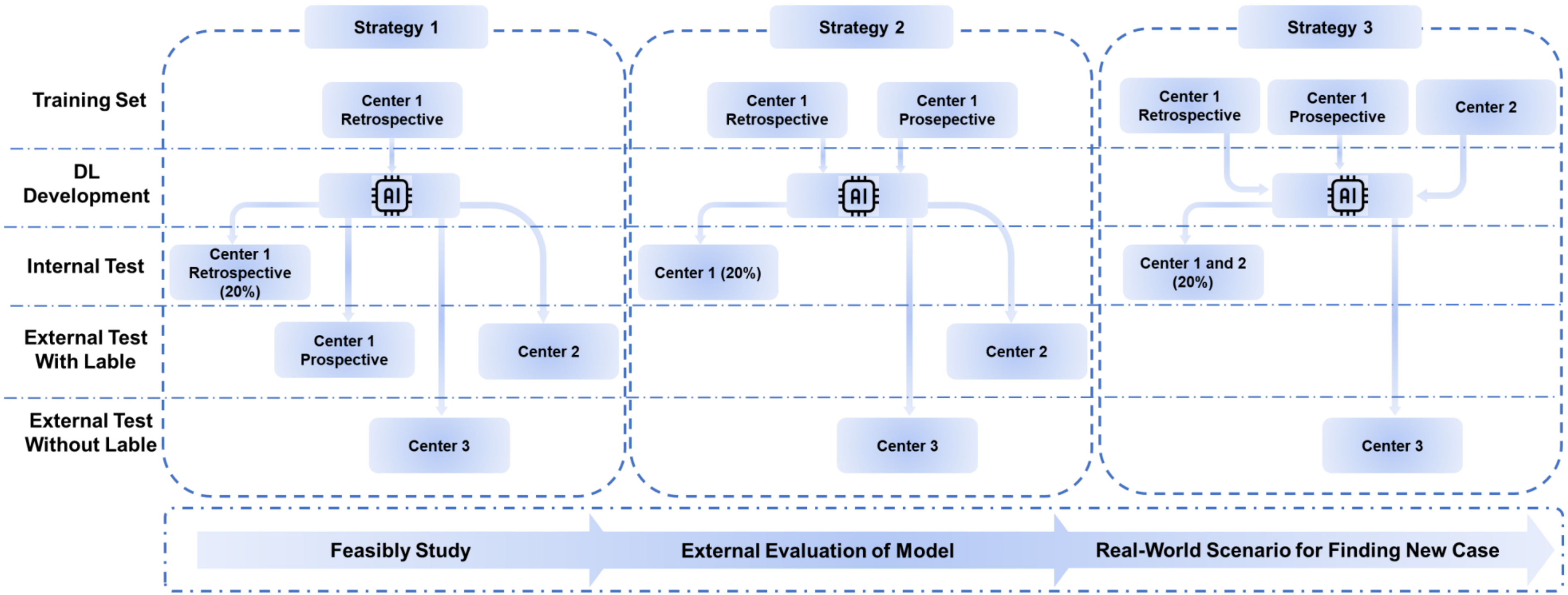

Supplemental Figure 27: Different possible strategies implemented to develop a more generalizable model using all available labeled datasets. The whole study was performed using Strategy 1; however, with Strategies 2 and 3, we expect the model to be more generalizable in real clinical scenarios and external test sets as more datasets will be used in the training set. Strategy 1: Model development was performed on the training dataset; the models were evaluated on the internal and external testing dataset with labeled cases. The external clinical testing dataset was used to evaluate the true and false positives, as the labeling was not available for this dataset. Strategy 2: Model training was performed on the entire dataset from Bern University Hospital. The labeled dataset from Zurich University Hospital served as an external testing dataset. The unlabeled open-access CCTA dataset (Guangdong Provincial People's Hospital) was used for external clinical evaluation, similar to Strategy 1. Strategy 3: Model training was performed on the entire datasets with labels, including data from Bern and Zurich University Hospitals. Following the previous strategy, external model performance was evaluated in the unlabeled dataset (external clinical evaluation dataset). Center 1: Bern University Hospital, Center 2: Zurich University Hospital, Center 3: Guangdong Provincial People's Hospital

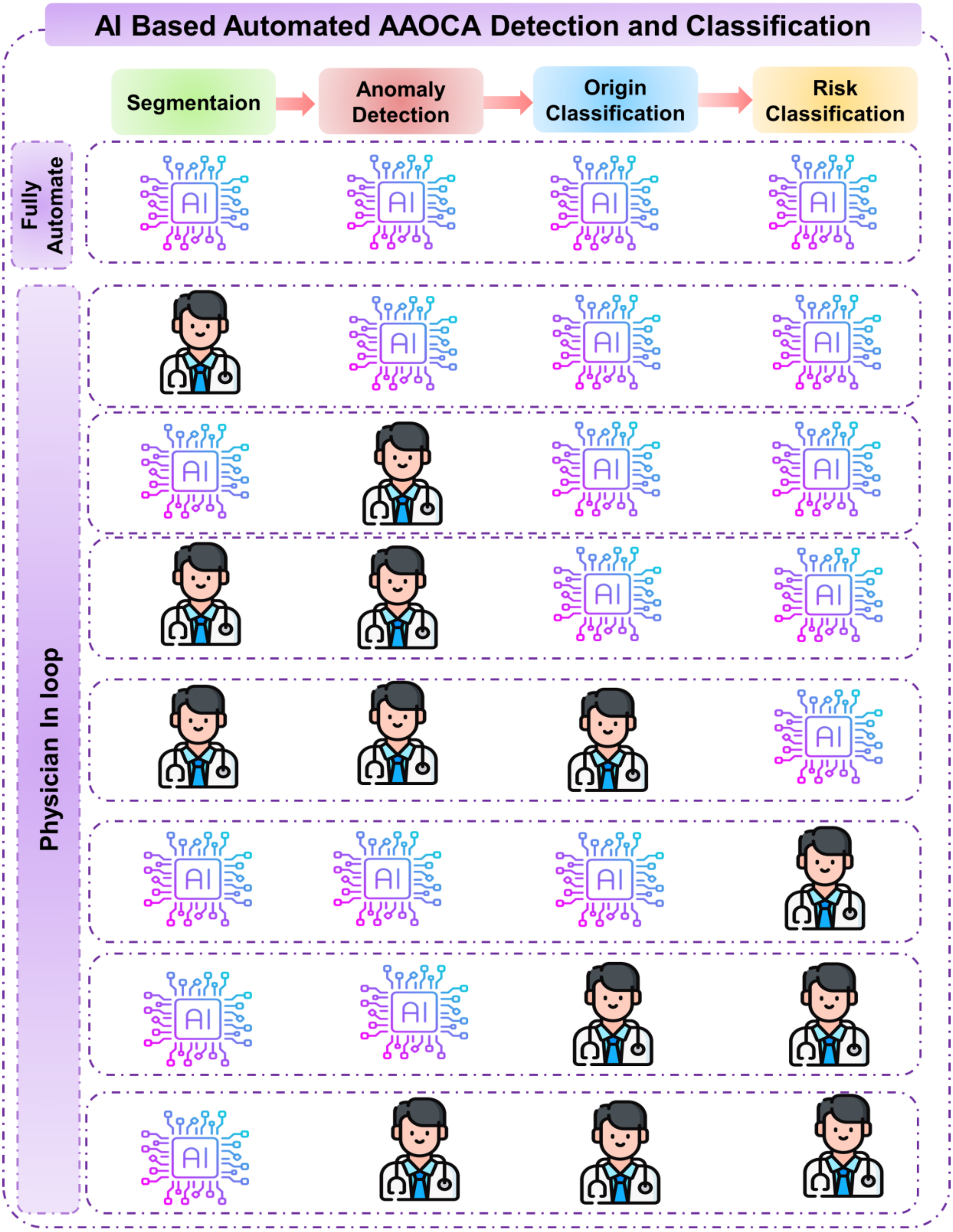

Supplemental Figure 28: Different possibilities for using the developed AI model in clinical scenarios include fully automated applications and physician-in-the-loop systems. Anomaly Detection: distinguishing between normal cases and those with AAOCA, Origin Classification: classifying the anomalous vessel into either the right (R-AAOCA) or left (L-AAOCA), Risk Classification: classifying the AAOCA risk, classifying it as either low-risk or high-risk.

Supplemental Tables

Supplemental Table 1: Summary statistics of the number of patients and images in each dataset for different classification tasks. Anomaly Detection: distinguishing between normal cases and those with AAOCA; Origin Classification: classifying the anomalous vessel into either the right (R-AAOCA) or left (L-AAOCA); risk Classification: classifying the AAOCA risk, classifying it as either low-risk or high-risk. Source data are provided as a Source Data file.

**Anomaly Detection**				
**Dataset**	**# All patients**	**# All images**	**#AAOCA patients**	**#AAOCA images**
**Training dataset**	536	1567	147	598
**Internal testing dataset**	359	1066	58	319
**External testing dataset**	483	497	130	139
**External clinical evaluation dataset**	998	998	Unknown	Unknown
**Origin Classification**				
	**# All patients**	**# All images**	**#L-AAOCA patients**	**# L-AAOCA images**
**Training dataset**	145	585	54	207
**Internal testing dataset**	57	309	15	48
**External testing dataset**	125	134	63	67
**Risk Classification**				
	**# All patients**	**# All images**	**#High-risk patients**	**# High risk-images**
**Training dataset**	144	582	107	465
**Internal testing dataset**	57	309	44	275
**External testing dataset**	125	134	74	80

Supplemental Table 2: Image-wise demographic information, scanner details, and image acquisition parameters across different datasets. This information was extracted from registry data and the DICOM headers of each image. Detailed information from the external clinical evaluation dataset is publicly available in[11]. Source data are provided as a Source Data file.

		Train dataset	Internal test dataset	External test dataset
**No.**		1567	1066	497
**Age, median [Q1, Q3]**		61.0 [54.0,67.0]	61.0 [54.0,68.0]	57.0 [50.0,64.0]
**Sex, no. (%)**	**Female**	559 (35.7)	349 (32.7)	172 (34.6)
	**Male**	1008 (64.3)	717 (67.3)	325 (65.4)
**BMI, median [Q1, Q3]**		25.6 [22.7,29.3]	25.6 [23.1,28.7]	25.9 [23.2,28.7]
**Manufacturer, n (%)**	**GE**	6 (0.4)		491 (98.8)
	**Siemens**	1521 (97.1)	1048 (98.3)	6 (1.2)
	**Canon**	10 (0.6)	3 (0.3)	
	**Philips**	19 (1.2)	13 (1.2)	
	**Toshiba**	11 (0.7)	2 (0.2)	
**Manufacturer Model Name, no. (%)**	**GE - Discovery CT750 HD**			21 (4.2)
	**GE - LightSpeed VCT**	1 (0.1)		20 (4.0)
	**GE - Revolution CT**	3 (0.2)		450 (90.5)
	**Siemens - SOMATOM Definition Flash**	1306 (83.3)	865 (81.1)	3 (0.6)
	**Siemens - Sensation 16**			1 (0.2)
	**Siemens - Sensation 64**			2 (0.4)
	**Canon - Aquilion ONE**	10 (0.6)	3 (0.3)	
	**Philips - Brilliance 64**	4 (0.3)	13 (1.2)	
	**Siemens - NAEOTOM Alpha**	180 (11.5)	170 (15.9)	
	**Siemens - SOMATOM Definition Edge**	17 (1.1)	5 (0.5)	
	**Siemens - SOMATOM Drive**		3 (0.3)	
	**Siemens - SOMATOM Force**		5 (0.5)	
	**Toshiba - Aquilion ONE**	11 (0.7)	2 (0.2)	
	**GE - Revolution EVO**	1 (0.1)		
	**GE - Revolution Frontier**	1 (0.1)		
	**Philips - Incisive CT**	5 (0.3)		
	**Philips - Spectral CT**	9 (0.6)		
	**Philips - iCT 256**	1 (0.1)		
	**Siemens - Perspective**	2 (0.1)		
	**Siemens - SOMATOM Definition AS**	10 (0.6)		
	**Siemens - Sensation 64 Cardiac**	6 (0.4)		
**Peak voltage (kVp), no. (%)**	**70**	2 (0.1)	4 (0.4)	
	**80**	33 (2.1)	30 (2.8)	23 (4.6)
	**100**	1051 (67.1)	591 (55.4)	368 (74.0)
	**110**	2 (0.1)		
	**120**	437 (27.9)	401 (37.6)	96 (19.3)
	**140**	42 (2.7)	40 (3.8)	10 (2.0)
**Exposure (mAs), median [Q1, Q3]**		196.1 [117.4,267.6]	209.0 [122.3,291.6]	84.0 [72.8,100.2]
**X-Spacing, median [Q1, Q3]**		0.4 [0.3,0.6]	0.4 [0.3,0.6]	0.5 [0.4,0.5]
**Y-Spacing, median [Q1, Q3]**		0.4 [0.3,0.6]	0.4 [0.3,0.6]	0.5 [0.4,0.5]
**Z-Spacing, median [Q1, Q3]**		0.7 [0.7,0.7]	0.7 [0.7,0.7]	0.6 [0.6,0.6]

The Bern dataset (used for training and internal testing) was collected between 2009 and 2024, while the Zurich dataset (used as an external test set) was collected between 2021 and 2023.

Supplemental Table 3: Patient-wise demographic information in different datasets**.** Detailed information from the external clinical evaluation dataset is publicly available in[11]. Source data are provided as a Source Data file.

		**Train dataset**	**Internal test dataset**	**External test dataset**
**No.**		536	359	483
**Age, median [Q1, Q3]**		61.0 [54.0,67.0]	62.0 [55.0,69.0]	57.0 [50.0,64.0]
**Sex, n (%)**	**Female**	200 (37.3)	126 (35.1)	165 (34.2)
	**Male**	336 (62.7)	233 (64.9)	318 (65.8)
**BMI, median [Q1, Q3]**		25.5 [23.0,28.7]	25.7 [23.4,29.1]	25.9 [23.2,28.7]

Supplemental Table 4: Summary of different classification metrics for the ensemble models for different test datasets in male population. Anomaly detection: distinguishing between normal cases and those with AAOCA; Origin classification: classifying the anomalous vessel into either the right (R-AAOCA) or left (L-AAOCA; Risk classification: scoring the AAOCA risk, classifying it as either low-risk or high-risk anatomy. AAOCA: Anomalous aortic origin of the coronary artery, ROC: Receiver Operating Characteristic, AUC: Area under the curve, F1-score: a measure of a test's accuracy, the harmonic means of precision and recall, PPV: Positive predictive value, AUPR: Area under the precision-recall curve. The slight differences in ROC-AUC values between figures and tables are due to the use of different libraries for calculations and rounding discrepancies. All values are rounded to three decimal places, which may result in a value of 1 (e.g., an AUC of 1.000 even if sensitivity and specificity are not both exactly 1.000). Source data are provided as a Source Data file.

	**Anomaly detection**		**Origin classification**		**Risk classification**	
	**Test internal**	**Test external**	**Test internal**	**Test external**	**Test internal**	**Test external**
**ROC AUC**	0.999	0.999	0.999	0.998	0.998	0.996
**Sensitivity**	0.988	0.952	0.889	0.979	0.986	0.983
**Specificity**	0.987	1	1	1	1	0.976
**F1-score**	0.981	0.975	0.941	0.989	0.993	0.983
**PPV**	0.975	1	1	1	1	0.983
**AUPR**	0.998	0.998	0.995	0.998	1	0.997
**Accuracy**	0.987	0.985	0.987	0.99	0.987	0.98

Supplemental Table 5: Summary of different classification metrics for the ensemble models for different test datasets in female population. Anomaly detection: distinguishing between normal cases and those with AAOCA; Origin classification: classifying the anomalous vessel into either the right (R-AAOCA) or left (L-AAOCA; Risk classification: scoring the AAOCA risk, classifying it as either low-risk or high-risk anatomy. AAOCA: Anomalous aortic origin of the coronary artery, ROC: Receiver Operating Characteristic, AUC: Area under the curve, F1-score: a measure of a test's accuracy, the harmonic means of precision and recall, PPV: Positive predictive value, AUPR: Area under the precision-recall curve. The slight differences in ROC-AUC values between figures and tables are due to the use of different libraries for calculations and rounding discrepancies. All values are rounded to three decimal places, which may result in a value of 1 (e.g., an AUC of 1.000 even if sensitivity and specificity are not both exactly 1.000). Source data are provided as a Source Data file.

	**Anomaly detection**		**Origin classification**		**Risk classification**	
	**Test internal**	**Test external**	**Test internal**	**Test external**	**Test internal**	**Test external**
**ROC AUC**	0.997	1	1	1	1	0.992
**Sensitivity**	0.987	0.971	1	0.9	1	0.9
**Specificity**	0.993	1	1	1	1	0.923
**F1-score**	0.981	0.986	1	0.947	1	0.923
**PPV**	0.975	1	1	1	1	0.947
**AUPR**	0.991	1	1	1	1	0.995
**Accuracy**	0.991	0.994	1	0.941	1	0.909

Supplemental Table 6: Summary of different classification metrics across different folds and mean and ensemble results from 5-fold in anomaly detection in different testing datasets. Anomaly Detection: distinguishing between normal cases and those with AAOCA. AAOCA: Anomalous aortic origin of the coronary artery, ROC: Receiver Operating Characteristic, AUC: Area under the curve, F1-score: a measure of a test's accuracy, the harmonic mean of precision and recall, PPV: Positive predictive value, AUPR: Area under the precision-recall curve. The slight differences in ROC-AUC values between figures and tables are due to the use of different libraries for calculations and rounding discrepancies. All values are rounded to three decimal places, which may result in a value of 1 (e.g., an AUC of 1.000 even if sensitivity and specificity are not both exactly 1.000). Source data are provided as a Source Data file.

Datasets	Metrics	Fold 1	Fold 2	Fold 3	Fold 4	Fold 5	Mean	Ensemble
**Internal Testing Dataset**	**ROC AUC**	0.993	0.99	0.994	0.994	0.995	0.993	0.998
	**Sensitivity**	0.978	0.925	0.984	0.956	0.994	0.967	0.987
	**Specificity**	0.984	0.992	0.979	0.987	0.976	0.983	0.989
	**F1-score**	0.97	0.952	0.968	0.962	0.969	0.964	0.981
	**PPV**	0.963	0.98	0.952	0.968	0.946	0.962	0.975
	**AUPR**	0.971	0.984	0.982	0.982	0.978	0.979	0.996
	**Accuracy**	0.982	0.972	0.98	0.977	0.981	0.979	0.989
**External Testing Dataset**	**ROC AUC**	0.994	0.997	0.999	0.999	0.998	0.997	0.999
	**Sensitivity**	0.928	0.942	0.95	0.964	0.971	0.951	0.957
	**Specificity**	0.994	0.994	0.994	0.997	0.98	0.992	1
	**F1-score**	0.956	0.963	0.967	0.978	0.961	0.965	0.978
	**PPV**	0.985	0.985	0.985	0.993	0.951	0.98	1
	**AUPR**	0.992	0.994	0.997	0.997	0.994	0.995	0.999
	**Accuracy**	0.976	0.98	0.982	0.988	0.978	0.981	0.988

Supplemental Table 7: Summary of different classification metrics across different folds and mean and ensemble results from 5-fold in anomaly detection in different testing datasets for the male population. Anomaly Detection: distinguishing between normal cases and those with AAOCA. AAOCA: Anomalous aortic origin of the coronary artery, ROC: Receiver Operating Characteristic, AUC: Area under the curve, F1-score: a measure of a test's accuracy, the harmonic mean of precision and recall, PPV: Positive predictive value, AUPR: Area under the precision-recall curve. The slight differences in ROC-AUC values between figures and tables are due to the use of different libraries for calculations and rounding discrepancies. All values are rounded to three decimal places, which may result in a value of 1 (e.g., an AUC of 1.000 even if sensitivity and specificity are not both exactly 1.000). Source data are provided as a Source Data file.

Datasets	Metrics	Fold 1	Fold 2	Fold 3	Fold 4	Fold 5	Mean	Ensemble
**Internal Testing Dataset**	**ROC AUC**	0.995	0.988	0.998	0.996	0.996	0.995	0.999
	**Sensitivity**	0.979	0.905	0.983	0.946	0.996	0.962	0.988
	**Specificity**	0.979	0.992	0.981	0.985	0.966	0.981	0.987
	**F1-score**	0.969	0.942	0.973	0.958	0.966	0.962	0.981
	**PPV**	0.959	0.982	0.963	0.97	0.938	0.962	0.975
	**AUPR**	0.98	0.982	0.996	0.991	0.988	0.987	0.998
	**Accuracy**	0.979	0.962	0.982	0.972	0.976	0.974	0.987
**External Testing Dataset**	**ROC AUC**	0.992	0.996	0.999	0.998	0.998	0.996	0.999
	**Sensitivity**	0.923	0.933	0.952	0.952	0.981	0.948	0.952
	**Specificity**	0.991	0.995	0.991	0.995	0.977	0.99	1
	**F1-score**	0.95	0.96	0.966	0.971	0.967	0.963	0.975
	**PPV**	0.98	0.99	0.98	0.99	0.953	0.979	1
	**AUPR**	0.99	0.993	0.997	0.995	0.996	0.994	0.998
	**Accuracy**	0.969	0.975	0.978	0.982	0.978	0.977	0.985

Supplemental Table 8: Summary of different classification metrics across different folds and mean and ensemble results from 5-fold in anomaly detection in different testing datasets for the female population. Anomaly Detection: distinguishing between normal cases and those with AAOCA. AAOCA: Anomalous aortic origin of the coronary artery, ROC: Receiver Operating Characteristic, AUC: Area under the curve, F1-score: a measure of a test's accuracy, the harmonic mean of precision and recall, PPV: Positive predictive value, AUPR: Area under the precision-recall curve. The slight differences in ROC-AUC values between figures and tables are due to the use of different libraries for calculations and rounding discrepancies. All values are rounded to three decimal places, which may result in a value of 1 (e.g., an AUC of 1.000 even if sensitivity and specificity are not both exactly 1.000). Source data are provided as a Source Data file.

Datasets	Metrics	Fold 1	Fold 2	Fold 3	Fold 4	Fold 5	Mean	Ensemble
**Internal Testing Dataset**	**ROC AUC**	0.989	0.991	0.985	0.991	0.993	0.99	0.997
	**Sensitivity**	0.974	0.987	0.987	0.987	0.987	0.985	0.987
	**Specificity**	0.993	0.993	0.974	0.989	0.993	0.988	0.993
	**F1-score**	0.974	0.981	0.951	0.975	0.981	0.972	0.981
	**PPV**	0.974	0.975	0.917	0.962	0.975	0.961	0.975
	**AUPR**	0.934	0.99	0.931	0.933	0.945	0.947	0.991
	**Accuracy**	0.989	0.991	0.977	0.989	0.991	0.987	0.991
**External Testing Dataset**	**ROC AUC**	1	1	0.999	1	0.997	0.999	1
	**Sensitivity**	0.943	0.971	0.943	1	0.943	0.96	0.971
	**Specificity**	1	0.993	1	1	0.985	0.996	1
	**F1-score**	0.971	0.971	0.971	1	0.943	0.971	0.986
	**PPV**	1	0.971	1	1	0.943	0.983	1
	**AUPR**	1	0.999	0.998	1	0.989	0.997	1
	**Accuracy**	0.988	0.988	0.988	1	0.977	0.988	0.994

Supplemental Table 9: Summary of different classification metrics across different folds and mean and ensemble results from 5-fold in anomalous coronary artery origin classification (R-AAOCA vs. L-AAOCA) in different testing datasets. Origin Classification: classifying the anomalous vessel into either the right (R-AAOCA) or left (L-AAOCA). AAOCA: Anomalous aortic origin of the coronary artery, ROC: Receiver Operating Characteristic, AUC: Area under the curve, F1-score: a measure of a test's accuracy, the harmonic mean of precision and recall, PPV: Positive predictive value, AUPR: Area under the precision-recall curve. The slight differences in ROC-AUC values between figures and tables are due to the use of different libraries for calculations and rounding discrepancies. All values are rounded to three decimal places, which may result in a value of 1 (e.g., an AUC of 1.000 even if sensitivity and specificity are not both exactly 1.000). Source data are provided as a Source Data file.

Datasets	Metrics	Fold 1	Fold 2	Fold 3	Fold 4	Fold 5	Mean	Ensemble
**Internal Testing Dataset**	**ROC AUC**	1	0.994	0.997	0.999	0.999	0.998	0.999
	**Sensitivity**	0.938	0.875	0.958	0.938	0.938	0.929	0.938
	**Specificity**	1	0.989	0.996	1	1	0.997	1
	**F1-score**	0.968	0.903	0.968	0.968	0.968	0.955	0.968
	**PPV**	1	0.933	0.979	1	1	0.982	1
	**AUPR**	0.999	0.976	0.988	0.996	0.995	0.991	0.997
	**Accuracy**	0.99	0.971	0.99	0.99	0.99	0.986	0.99
**External Testing Dataset**	**ROC AUC**	0.99	0.988	0.997	0.978	0.997	0.99	0.999
	**Sensitivity**	0.97	0.955	0.94	0.94	0.985	0.958	0.955
	**Specificity**	1	0.985	1	0.97	0.985	0.988	1
	**F1-score**	0.985	0.97	0.969	0.955	0.985	0.973	0.977
	**PPV**	1	0.985	1	0.969	0.985	0.988	1
	**AUPR**	0.994	0.992	0.997	0.984	0.997	0.993	0.999
	**Accuracy**	0.985	0.97	0.97	0.955	0.985	0.973	0.978

Supplemental Table 10: Summary of different classification metrics across different folds and mean and ensemble results from 5-fold in anomalous coronary artery origin classification (R-AAOCA vs. L-AAOCA) in different testing datasets for the male population. Origin Classification: classifying the anomalous vessel into either the right (R-AAOCA) or left (L-AAOCA). AAOCA: Anomalous aortic origin of the coronary artery, ROC: Receiver Operating Characteristic, AUC: Area under the curve, F1-score: a measure of a test's accuracy, the harmonic mean of precision and recall, PPV: Positive predictive value, AUPR: Area under the precision-recall curve. The slight differences in ROC-AUC values between figures and tables are due to the use of different libraries for calculations and rounding discrepancies. All values are rounded to three decimal places, which may result in a value of 1 (e.g., an AUC of 1.000 even if sensitivity and specificity are not both exactly 1.000). Source data are provided as a Source Data file.

Datasets	Metrics	Fold 1	Fold 2	Fold 3	Fold 4	Fold 5	Mean	Ensemble
**Internal Testing Dataset**	**ROC AUC**	0.999	0.991	0.994	0.999	0.999	0.996	0.999
	**Sensitivity**	0.889	0.815	0.926	0.889	0.889	0.881	0.889
	**Specificity**	1	1	0.995	1	1	0.999	1
	**F1-score**	0.941	0.898	0.943	0.941	0.941	0.933	0.941
	**PPV**	1	1	0.962	1	1	0.992	1
	**AUPR**	0.996	0.956	0.972	0.992	0.99	0.981	0.995
	**Accuracy**	0.987	0.978	0.987	0.987	0.987	0.985	0.987
**External Testing Dataset**	**ROC AUC**	0.986	0.998	0.996	0.991	0.996	0.994	0.998
	**Sensitivity**	0.957	0.979	0.957	0.957	0.979	0.966	0.979
	**Specificity**	1	0.981	1	0.962	0.981	0.985	1
	**F1-score**	0.978	0.979	0.978	0.957	0.979	0.974	0.989
	**PPV**	1	0.979	1	0.957	0.979	0.983	1
	**AUPR**	0.991	0.998	0.997	0.991	0.996	0.994	0.998
	**Accuracy**	0.98	0.98	0.98	0.96	0.98	0.976	0.99

Supplemental Table 11: Summary of different classification metrics across different folds and mean and ensemble results from 5-fold in anomalous coronary artery origin classification (R-AAOCA vs. L-AAOCA) in different testing datasets for the female population. Origin Classification: classifying the anomalous vessel into either the right (R-AAOCA) or left (L-AAOCA). AAOCA: Anomalous aortic origin of the coronary artery, ROC: Receiver Operating Characteristic, AUC: Area under the curve, F1-score: a measure of a test's accuracy, the harmonic mean of precision and recall, PPV: Positive predictive value, AUPR: Area under the precision-recall curve. The slight differences in ROC-AUC values between figures and tables are due to the use of different libraries for calculations and rounding discrepancies. All values are rounded to three decimal places, which may result in a value of 1 (e.g., an AUC of 1.000 even if sensitivity and specificity are not both exactly 1.000). Source data are provided as a Source Data file.

Datasets	Metrics	Fold 1	Fold 2	Fold 3	Fold 4	Fold 5	Mean	Ensemble
**Internal Testing Dataset**	**ROC AUC**	1	0.997	1	1	1	0.999	1
	**Sensitivity**	1	0.952	1	1	1	0.99	1
	**Specificity**	1	0.946	1	1	1	0.989	1
	**F1-score**	1	0.909	1	1	1	0.982	1
	**PPV**	1	0.87	1	1	1	0.974	1
	**AUPR**	1	0.994	1	1	1	0.999	1
	**Accuracy**	1	0.948	1	1	1	0.99	1
**External Testing Dataset**	**ROC AUC**	1	0.975	1	0.946	1	0.984	1
	**Sensitivity**	1	0.9	0.9	0.9	1	0.94	0.9
	**Specificity**	1	1	1	1	1	1	1
	**F1-score**	1	0.947	0.947	0.947	1	0.968	0.947
	**PPV**	1	1	1	1	1	1	1
	**AUPR**	1	0.987	1	0.973	1	0.992	1
	**Accuracy**	1	0.941	0.941	0.941	1	0.965	0.941

Supplemental Table 12: Summary of different classification metrics, including mean and ensemble results from 5-fold in risk classifying (high risk vs. low risk) in different testing datasets (Risk Classification). Risk Classification: classifying the AAOCA risk, classifying it as either low-risk or high-risk. AAOCA: Anomalous aortic origin of the coronary artery, ROC: Receiver Operating Characteristic, AUC: Area under the curve, F1-score: a measure of a test's accuracy, the harmonic mean of precision and recall, PPV: Positive predictive value, AUPR: Area under the precision-recall curve. The slight differences in ROC-AUC values between figures and tables are due to the use of different libraries for calculations and rounding discrepancies. All values are rounded to three decimal places, which may result in a value of 1 (e.g., an AUC of 1.000 even if sensitivity and specificity are not both exactly 1.000). Source data are provided as a Source Data file.

Datasets	Metrics	Fold 1	Fold 2	Fold 3	Fold 4	Fold 5	Mean	Ensemble
**Internal Testing Dataset**	**ROC AUC**	0.994	0.984	1	1	1	0.996	0.999
	**Sensitivity**	0.982	0.902	0.996	0.967	0.978	0.965	0.989
	**Specificity**	1	1	1	1	1	1	1
	**F1-score**	0.991	0.948	0.998	0.983	0.989	0.982	0.995
	**PPV**	1	1	1	1	1	1	1
	**AUPR**	0.999	0.998	1	1	1	0.999	1
	**Accuracy**	0.984	0.913	0.997	0.971	0.981	0.969	0.99
**External Testing Dataset**	**ROC AUC**	0.989	0.98	0.999	0.998	0.997	0.993	0.996
	**Sensitivity**	0.975	0.875	0.975	0.95	0.975	0.95	0.962
	**Specificity**	0.963	0.944	0.981	0.963	0.963	0.963	0.963
	**F1-score**	0.975	0.915	0.981	0.962	0.975	0.962	0.969
	**PPV**	0.975	0.959	0.987	0.974	0.975	0.974	0.975
	**AUPR**	0.992	0.985	0.999	0.999	0.998	0.995	0.997
	**Accuracy**	0.97	0.903	0.978	0.955	0.97	0.955	0.963

Supplemental Table 13: Summary of different classification metrics, including mean and ensemble results from 5-fold in risk classifying (high risk vs. low risk) in different testing datasets (Risk Classification) for the male population. Risk Classification: classifying the AAOCA risk, classifying it as either low-risk or high-risk. AAOCA: Anomalous aortic origin of the coronary artery, ROC: Receiver Operating Characteristic, AUC: Area under the curve, F1-score: a measure of a test's accuracy, the harmonic mean of precision and recall, PPV: Positive predictive value, AUPR: Area under the precision-recall curve. The slight differences in ROC-AUC values between figures and tables are due to the use of different libraries for calculations and rounding discrepancies. All values are rounded to three decimal places, which may result in a value of 1 (e.g., an AUC of 1.000 even if sensitivity and specificity are not both exactly 1.000). Source data are provided as a Source Data file.

Datasets	Metrics	Fold 1	Fold 2	Fold 3	Fold 4	Fold 5	Mean	Ensemble
**Internal Testing Dataset**	**ROC AUC**	0.993	0.969	1	0.999	1	0.992	0.998
	**Sensitivity**	0.977	0.883	0.995	0.958	0.972	0.957	0.986
	**Specificity**	1	1	1	1	1	1	1
	**F1-score**	0.988	0.938	0.998	0.979	0.986	0.978	0.993
	**PPV**	1	1	1	1	1	1	1
	**AUPR**	0.999	0.997	1	1	1	0.999	1
	**Accuracy**	0.978	0.892	0.996	0.961	0.974	0.96	0.987
**External Testing Dataset**	**ROC AUC**	0.997	0.98	0.999	0.999	0.997	0.994	0.996
	**Sensitivity**	0.983	0.867	0.983	0.95	0.983	0.953	0.983
	**Specificity**	0.976	0.951	0.976	0.976	0.976	0.971	0.976
	**F1-score**	0.983	0.912	0.983	0.966	0.983	0.966	0.983
	**PPV**	0.983	0.963	0.983	0.983	0.983	0.979	0.983
	**AUPR**	0.998	0.986	0.999	0.999	0.998	0.996	0.997
	**Accuracy**	0.98	0.901	0.98	0.96	0.98	0.96	0.98

Supplemental Table 14: Summary of different classification metrics, including mean and ensemble results from 5-fold in risk classifying (high risk vs. low risk) in different testing datasets (Risk Classification) for the female population. Risk Classification: classifying the AAOCA risk, classifying it as either low-risk or high-risk. AAOCA: Anomalous aortic origin of the coronary artery, ROC: Receiver Operating Characteristic, AUC: Area under the curve, F1-score: a measure of a test's accuracy, the harmonic mean of precision and recall, PPV: Positive predictive value, AUPR: Area under the precision-recall curve. The slight differences in ROC-AUC values between figures and tables are due to the use of different libraries for calculations and rounding discrepancies. All values are rounded to three decimal places, which may result in a value of 1 (e.g., an AUC of 1.000 even if sensitivity and specificity are not both exactly 1.000). Source data are provided as a Source Data file.

Datasets	Metrics	Fold 1	Fold 2	Fold 3	Fold 4	Fold 5	Mean	Ensemble
**Internal Testing Dataset**	**ROC AUC**	1	1	1	1	1	1	1
	**Sensitivity**	1	0.967	1	1	1	0.993	1
	**Specificity**	1	1	1	1	1	1	1
	**F1-score**	1	0.983	1	1	1	0.997	1
	**PPV**	1	1	1	1	1	1	1
	**AUPR**	1	1	1	1	1	1	1
	**Accuracy**	1	0.974	1	1	1	0.995	1
**External Testing Dataset**	**ROC AUC**	0.946	0.988	1	0.996	0.996	0.985	0.992
	**Sensitivity**	0.95	0.9	0.95	0.95	0.95	0.94	0.9
	**Specificity**	0.923	0.923	1	0.923	0.923	0.938	0.923
	**F1-score**	0.95	0.923	0.974	0.95	0.95	0.949	0.923
	**PPV**	0.95	0.947	1	0.95	0.95	0.959	0.947
	**AUPR**	0.956	0.992	1	0.998	0.998	0.989	0.995
	**Accuracy**	0.939	0.909	0.97	0.939	0.939	0.939	0.909

Supplemental Table 15: Summary of various classification metrics for the ensemble model with different cut-offs in anomaly detection in different testing datasets. Anomaly Detection: distinguishing between normal cases and those with AAOCA. AAOCA: Anomalous aortic origin of the coronary artery, F1-score: a measure of a test's accuracy, the harmonic mean of precision and recall, PPV: Positive predictive value, AUPR: Area under the precision-recall curve. The slight differences in ROC-AUC values between figures and tables are due to the use of different libraries for calculations and rounding discrepancies. All values are rounded to three decimal places, which may result in a value of 1 (e.g., an AUC of 1.000 even if sensitivity and specificity are not both exactly 1.000). Source data are provided as a Source Data file.

Datasets	Cut-off	Sensitivity	Specificity	F1-score	PPV	Accuracy
**Internal Testing Dataset**	0.1	0.997	0.953	0.946	0.901	0.966
	0.2	0.997	0.973	0.968	0.941	0.98
	0.3	0.997	0.987	0.983	0.97	0.99
	0.4	0.987	0.988	0.98	0.972	0.988
	0.5	0.987	0.989	0.981	0.975	0.989
	0.6	0.966	0.991	0.972	0.978	0.983
	0.7	0.947	0.993	0.965	0.984	0.979
	0.8	0.915	0.996	0.951	0.99	0.972
	0.9	0.909	0.996	0.948	0.99	0.97
**External Testing Dataset**	0.1	0.993	0.975	0.965	0.939	0.98
	0.2	0.993	0.986	0.979	0.965	0.988
	0.3	0.986	0.986	0.975	0.965	0.986
	0.4	0.971	0.997	0.982	0.993	0.99
	0.5	0.957	1	0.978	1	0.988
	0.6	0.942	1	0.97	1	0.984
	0.7	0.928	1	0.963	1	0.98
	0.8	0.914	1	0.955	1	0.976
	0.9	0.899	1	0.947	1	0.972

Supplemental Table 16: Summary of various classification metrics for the ensemble model with different cut-offs in anomaly detection in different testing datasets for the male population. Anomaly Detection: distinguishing between normal cases and those with AAOCA. AAOCA: Anomalous aortic origin of the coronary artery, F1-score: a measure of a test's accuracy, the harmonic mean of precision and recall, PPV: Positive predictive value, AUPR: Area under the precision-recall curve. The slight differences in ROC-AUC values between figures and tables are due to the use of different libraries for calculations and rounding discrepancies. All values are rounded to three decimal places, which may result in a value of 1 (e.g., an AUC of 1.000 even if sensitivity and specificity are not both exactly 1.000). Source data are provided as a Source Data file.

Datasets	Cut-off	Sensitivity	Specificity	F1-score	PPV	Accuracy
**Internal Testing Dataset**	**0.1**	1	0.943	0.947	0.899	0.962
	**0.2**	1	0.966	0.968	0.938	0.978
	**0.3**	1	0.983	0.984	0.968	0.989
	**0.4**	0.988	0.985	0.979	0.971	0.986
	**0.5**	0.988	0.987	0.981	0.975	0.987
	**0.6**	0.959	0.989	0.969	0.979	0.979
	**0.7**	0.934	0.994	0.959	0.987	0.974
	**0.8**	0.896	0.998	0.943	0.995	0.964
	**0.9**	0.888	0.998	0.939	0.995	0.961
**External Testing Dataset**	**0.1**	0.99	0.968	0.963	0.936	0.975
	**0.2**	0.99	0.982	0.976	0.963	0.985
	**0.3**	0.981	0.982	0.971	0.962	0.982
	**0.4**	0.971	0.995	0.981	0.99	0.988
	**0.5**	0.952	1	0.975	1	0.985
	**0.6**	0.933	1	0.965	1	0.978
	**0.7**	0.923	1	0.96	1	0.975
	**0.8**	0.913	1	0.955	1	0.972
	**0.9**	0.904	1	0.949	1	0.969

Supplemental Table 17: Summary of various classification metrics for the ensemble model with different cut-offs in anomaly detection in different testing datasets for the female population. Anomaly Detection: distinguishing between normal cases and those with AAOCA. AAOCA: Anomalous aortic origin of the coronary artery, F1-score: a measure of a test's accuracy, the harmonic mean of precision and recall, PPV: Positive predictive value, AUPR: Area under the precision-recall curve. The slight differences in ROC-AUC values between figures and tables are due to the use of different libraries for calculations and rounding discrepancies. All values are rounded to three decimal places, which may result in a value of 1 (e.g., an AUC of 1.000 even if sensitivity and specificity are not both exactly 1.000). Source data are provided as a Source Data file.

Datasets	Cut-off	Sensitivity	Specificity	F1-score	PPV	Accuracy
**Internal Testing Dataset**	**0.1**	0.987	0.97	0.945	0.906	0.974
	**0.2**	0.987	0.985	0.969	0.951	0.986
	**0.3**	0.987	0.993	0.981	0.975	0.991
	**0.4**	0.987	0.993	0.981	0.975	0.991
	**0.5**	0.987	0.993	0.981	0.975	0.991
	**0.6**	0.987	0.993	0.981	0.975	0.991
	**0.7**	0.987	0.993	0.981	0.975	0.991
	**0.8**	0.974	0.993	0.974	0.974	0.989
	**0.9**	0.974	0.993	0.974	0.974	0.989
**External Testing Dataset**	**0.1**	1	0.985	0.972	0.946	0.988
	**0.2**	1	0.993	0.986	0.972	0.994
	**0.3**	1	0.993	0.986	0.972	0.994
	**0.4**	0.971	1	0.986	1	0.994
	**0.5**	0.971	1	0.986	1	0.994
	**0.6**	0.971	1	0.986	1	0.994
	**0.7**	0.943	1	0.971	1	0.988
	**0.8**	0.914	1	0.955	1	0.983
	**0.9**	0.886	1	0.939	1	0.977

Supplemental Table 18: Summary of various classification metrics for the ensemble model with different cut-offs in anomalous coronary artery origin classification (R-AAOCA vs. L-AAOCA) in different testing datasets. Origin Classification: classifying the anomalous vessel into either the right (R-AAOCA) or left (L-AAOCA). AAOCA: Anomalous aortic origin of the coronary artery, F1-score: a measure of a test's accuracy, the harmonic mean of precision and recall, PPV: Positive predictive value, AUPR: Area under the precision-recall curve. The slight differences in ROC-AUC values between figures and tables are due to the use of different libraries for calculations and rounding discrepancies. All values are rounded to three decimal places, which may result in a value of 1 (e.g., an AUC of 1.000 even if sensitivity and specificity are not both exactly 1.000). Source data are provided as a Source Data file.

Datasets	Cut-off	Sensitivity	Specificity	F1-score	PPV	Accuracy
**Internal Testing Dataset**	**0.1**	0.979	0.985	0.949	0.922	0.984
	**0.2**	0.979	0.992	0.969	0.959	0.99
	**0.3**	0.958	1	0.979	1	0.994
	**0.4**	0.938	1	0.968	1	0.99
	**0.5**	0.938	1	0.968	1	0.99
	**0.6**	0.938	1	0.968	1	0.99
	**0.7**	0.917	1	0.957	1	0.987
	**0.8**	0.875	1	0.933	1	0.981
	**0.9**	0.875	1	0.933	1	0.981
**External Testing Dataset**	**0.1**	0.985	0.94	0.964	0.943	0.963
	**0.2**	0.985	0.955	0.971	0.957	0.97
	**0.3**	0.985	0.985	0.985	0.985	0.985
	**0.4**	0.97	1	0.985	1	0.985
	**0.5**	0.955	1	0.977	1	0.978
	**0.6**	0.955	1	0.977	1	0.978
	**0.7**	0.94	1	0.969	1	0.97
	**0.8**	0.925	1	0.961	1	0.963
	**0.9**	0.91	1	0.953	1	0.955

Supplemental Table 19: Summary of various classification metrics for the ensemble model with different cut-offs in anomalous coronary artery origin classification (R-AAOCA vs. L-AAOCA) in different testing datasets for the male population. Origin Classification: classifying the anomalous vessel into either the right (R-AAOCA) or left (L-AAOCA). AAOCA: Anomalous aortic origin of the coronary artery, F1-score: a measure of a test's accuracy, the harmonic mean of precision and recall, PPV: Positive predictive value, AUPR: Area under the precision-recall curve. The slight differences in ROC-AUC values between figures and tables are due to the use of different libraries for calculations and rounding discrepancies. All values are rounded to three decimal places, which may result in a value of 1 (e.g., an AUC of 1.000 even if sensitivity and specificity are not both exactly 1.000). Source data are provided as a Source Data file.

Datasets	Cut-off	Sensitivity	Specificity	F1-score	PPV	Accuracy
**Internal Testing Dataset**	**0.1**	0.963	0.995	0.963	0.963	0.991
	**0.2**	0.963	0.995	0.963	0.963	0.991
	**0.3**	0.926	1	0.962	1	0.991
	**0.4**	0.889	1	0.941	1	0.987
	**0.5**	0.889	1	0.941	1	0.987
	**0.6**	0.889	1	0.941	1	0.987
	**0.7**	0.852	1	0.92	1	0.983
	**0.8**	0.815	1	0.898	1	0.978
	**0.9**	0.815	1	0.898	1	0.978
**External Testing Dataset**	**0.1**	0.979	0.925	0.948	0.92	0.95
	**0.2**	0.979	0.943	0.958	0.939	0.96
	**0.3**	0.979	0.981	0.979	0.979	0.98
	**0.4**	0.979	1	0.989	1	0.99
	**0.5**	0.979	1	0.989	1	0.99
	**0.6**	0.979	1	0.989	1	0.99
	**0.7**	0.957	1	0.978	1	0.98
	**0.8**	0.936	1	0.967	1	0.97
	**0.9**	0.915	1	0.956	1	0.96

Supplemental Table 20: Summary of various classification metrics for the ensemble model with different cut-offs in anomalous coronary artery origin classification (R-AAOCA vs. L-AAOCA) in different testing datasets for the female population. Origin Classification: classifying the anomalous vessel into either the right (R-AAOCA) or left (L-AAOCA). AAOCA: Anomalous aortic origin of the coronary artery, F1-score: a measure of a test's accuracy, the harmonic mean of precision and recall, PPV: Positive predictive value, AUPR: Area under the precision-recall curve. The slight differences in ROC-AUC values between figures and tables are due to the use of different libraries for calculations and rounding discrepancies. All values are rounded to three decimal places, which may result in a value of 1 (e.g., an AUC of 1.000 even if sensitivity and specificity are not both exactly 1.000). Source data are provided as a Source Data file.

Datasets	Cut-off	Sensitivity	Specificity	F1-score	PPV	Accuracy
**Internal Testing Dataset**	**0.1**	1	0.946	0.933	0.875	0.961
	**0.2**	1	0.982	0.977	0.955	0.987
	**0.3**	1	1	1	1	1
	**0.4**	1	1	1	1	1
	**0.5**	1	1	1	1	1
	**0.6**	1	1	1	1	1
	**0.7**	1	1	1	1	1
	**0.8**	0.952	1	0.976	1	0.987
	**0.9**	0.952	1	0.976	1	0.987
**External Testing Dataset**	**0.1**	1	1	1	1	1
	**0.2**	1	1	1	1	1
	**0.3**	1	1	1	1	1
	**0.4**	0.95	1	0.974	1	0.971
	**0.5**	0.9	1	0.947	1	0.941
	**0.6**	0.9	1	0.947	1	0.941
	**0.7**	0.9	1	0.947	1	0.941
	**0.8**	0.9	1	0.947	1	0.941
	**0.9**	0.9	1	0.947	1	0.941

Supplemental Table 21: Summary of various classification metrics for the ensemble model with different cut-offs in risk classification (high risk vs. low risk) in different testing datasets. Risk Classification: classifying the AAOCA risk, classifying it as either low-risk or high-risk. AAOCA: Anomalous aortic origin of the coronary artery, F1-score: a measure of a test's accuracy, the harmonic mean of precision and recall, PPV: Positive predictive value, AUPR: Area under the precision-recall curve. The slight differences in ROC-AUC values between figures and tables are due to the use of different libraries for calculations and rounding discrepancies. All values are rounded to three decimal places, which may result in a value of 1 (e.g., an AUC of 1.000 even if sensitivity and specificity are not both exactly 1.000). Source data are provided as a Source Data file.

Datasets	Cut-off	Sensitivity	Specificity	F1-score	PPV	Accuracy
**Internal Testing Dataset**	**0.1**	0.996	1	0.998	1	0.997
	**0.2**	0.996	1	0.998	1	0.997
	**0.3**	0.996	1	0.998	1	0.997
	**0.4**	0.993	1	0.996	1	0.994
	**0.5**	0.989	1	0.995	1	0.99
	**0.6**	0.985	1	0.993	1	0.987
	**0.7**	0.949	1	0.974	1	0.955
	**0.8**	0.935	1	0.966	1	0.942
	**0.9**	0.898	1	0.946	1	0.909
**External Testing Dataset**	**0.1**	0.988	0.944	0.975	0.963	0.97
	**0.2**	0.988	0.963	0.981	0.975	0.978
	**0.3**	0.988	0.963	0.981	0.975	0.978
	**0.4**	0.988	0.963	0.981	0.975	0.978
	**0.5**	0.962	0.963	0.969	0.975	0.963
	**0.6**	0.962	0.963	0.969	0.975	0.963
	**0.7**	0.938	0.963	0.955	0.974	0.948
	**0.8**	0.925	0.981	0.955	0.987	0.948
	**0.9**	0.875	0.981	0.927	0.986	0.918

Supplemental Table 22: Summary of various classification metrics for the ensemble model with different cut-offs in risk classification (high risk vs. low risk) in different testing datasets for male population. Risk Classification: classifying the AAOCA risk, classifying it as either low-risk or high-risk. AAOCA: Anomalous aortic origin of the coronary artery, F1-score: a measure of a test's accuracy, the harmonic mean of precision and recall, PPV: Positive predictive value, AUPR: Area under the precision-recall curve. The slight differences in ROC-AUC values between figures and tables are due to the use of different libraries for calculations and rounding discrepancies. All values are rounded to three decimal places, which may result in a value of 1 (e.g., an AUC of 1.000 even if sensitivity and specificity are not both exactly 1.000). Source data are provided as a Source Data file.

Datasets	Cut-off	Sensitivity	Specificity	F1-score	PPV	Accuracy
**Internal Testing Dataset**	**0.1**	0.995	1	0.998	1	0.996
	**0.2**	0.995	1	0.998	1	0.996
	**0.3**	0.995	1	0.998	1	0.996
	**0.4**	0.991	1	0.995	1	0.991
	**0.5**	0.986	1	0.993	1	0.987
	**0.6**	0.981	1	0.991	1	0.983
	**0.7**	0.935	1	0.966	1	0.94
	**0.8**	0.916	1	0.956	1	0.922
	**0.9**	0.879	1	0.935	1	0.888
**External Testing Dataset**	**0.1**	0.983	0.951	0.975	0.967	0.97
	**0.2**	0.983	0.976	0.983	0.983	0.98
	**0.3**	0.983	0.976	0.983	0.983	0.98
	**0.4**	0.983	0.976	0.983	0.983	0.98
	**0.5**	0.983	0.976	0.983	0.983	0.98
	**0.6**	0.983	0.976	0.983	0.983	0.98
	**0.7**	0.95	0.976	0.966	0.983	0.96
	**0.8**	0.933	0.976	0.957	0.982	0.95
	**0.9**	0.867	0.976	0.92	0.981	0.911

Supplemental Table 23: Summary of various classification metrics for the ensemble model with different cut-offs in risk classification (high risk vs. low risk) in different testing datasets for female population. Risk Classification: classifying the AAOCA risk, classifying it as either low-risk or high-risk. AAOCA: Anomalous aortic origin of the coronary artery, F1-score: a measure of a test's accuracy, the harmonic mean of precision and recall, PPV: Positive predictive value, AUPR: Area under the precision-recall curve. The slight differences in ROC-AUC values between figures and tables are due to the use of different libraries for calculations and rounding discrepancies. All values are rounded to three decimal places, which may result in a value of 1 (e.g., an AUC of 1.000 even if sensitivity and specificity are not both exactly 1.000). Source data are provided as a Source Data file.

Datasets	Cut-off	Sensitivity	Specificity	F1-score	PPV	Accuracy
**Internal Testing Dataset**	**0.1**	1	1	1	1	1
	**0.2**	1	1	1	1	1
	**0.3**	1	1	1	1	1
	**0.4**	1	1	1	1	1
	**0.5**	1	1	1	1	1
	**0.6**	1	1	1	1	1
	**0.7**	1	1	1	1	1
	**0.8**	1	1	1	1	1
	**0.9**	0.967	1	0.983	1	0.974
**External Testing Dataset**	**0.1**	1	0.923	0.976	0.952	0.97
	**0.2**	1	0.923	0.976	0.952	0.97
	**0.3**	1	0.923	0.976	0.952	0.97
	**0.4**	1	0.923	0.976	0.952	0.97
	**0.5**	0.9	0.923	0.923	0.947	0.909
	**0.6**	0.9	0.923	0.923	0.947	0.909
	**0.7**	0.9	0.923	0.923	0.947	0.909
	**0.8**	0.9	1	0.947	1	0.939
	**0.9**	0.9	1	0.947	1	0.939

Supplementary References

1 Tejani, A. S. et al. Checklist for Artificial Intelligence in Medical Imaging (CLAIM): 2024 Update. Radiol Artif Intell **6**, e240300 (2024). https://doi.org/10.1148/ryai.240300

2 Sounderajah, V. et al. Developing a reporting guideline for artificial intelligence-centred diagnostic test accuracy studies: the STARD-AI protocol. BMJ Open **11**, e047709 (2021). https://doi.org/10.1136/bmjopen-2020-047709

3 Hernandez-Boussard, T., Bozkurt, S., Ioannidis, J. P. A. & Shah, N. H. MINIMAR (MINimum Information for Medical AI Reporting): Developing reporting standards for artificial intelligence in health care. J Am Med Inform Assoc **27**, 2011-2015 (2020). https://doi.org/10.1093/jamia/ocaa088

4 McCormick, M., Liu, X., Jomier, J., Marion, C. & Ibanez, L. ITK: enabling reproducible research and open science. Front Neuroinform **8**, 13 (2014). https://doi.org/10.3389/fninf.2014.00013

5 Yoo, T. S. et al. Engineering and algorithm design for an image processing Api: a technical report on ITK--the Insight Toolkit. Stud Health Technol Inform **85**, 586-592 (2002).

6 Ansel, J. et al. in Proceedings of the 29th ACM International Conference on Architectural Support for Programming Languages and Operating Systems, Volume 2. 929-947.

7 Pérez-García, F., Sparks, R. & Ourselin, S. TorchIO: A Python library for efficient loading, preprocessing, augmentation and patch-based sampling of medical images in deep learning. Comput Methods Programs Biomed **208**, 106236 (2021). https://doi.org/10.1016/j.cmpb.2021.106236

8 Cardoso, M. J. et al. Monai: An open-source framework for deep learning in healthcare. arXiv preprint arXiv:2211.02701 (2022).

9 Van der Maaten, L. & Hinton, G. Visualizing data using t-SNE. Journal of machine learning research **9** (2008).

10 He, K., Zhang, X., Ren, S. & Sun, J. in Proceedings of the IEEE international conference on computer vision. 1026-1034.

11 Zeng, A. et al. ImageCAS: A large-scale dataset and benchmark for coronary artery segmentation based on computed tomography angiography images. Comput Med Imaging Graph **109**, 102287 (2023). https://doi.org/10.1016/j.compmedimag.2023.102287
