# Supplementary material for: AI-based detection and classification of anomalous aortic origin of coronary arteries using coronary CT angiography images
Source: Nat Commun. 2025 Apr 1;16:3095. doi: 10.1038/s41467-025-58362-9 (PMC11961624; doi:10.1038/s41467-025-58362-9)
Supplement: Supplementary file 2 — Reporting Summary [file 41467_2025_58362_MOESM2_ESM.pdf]

nature portfolio

Corresponding author(s): Prof. Dr. Dr Christoph Gräni

Last updated by author(s): Mar 4, 2025

Reporting Summary

Nature Portfolio wishes to improve the reproducibility of the work that we publish. This form provides structure for consistency and transparency in reporting. For further information on Nature Portfolio policies, see our Editorial Policies and the Editorial Policy Checklist.

Statistics

For all statistical analyses, confirm that the following items are present in the figure legend, table legend, main text, or Methods section.

n/a	Confirmed	
☐	☒	The exact sample size (*n*) for each experimental group/condition, given as a discrete number and unit of measurement
☒	☐	A statement on whether measurements were taken from distinct samples or whether the same sample was measured repeatedly
☒	☐	The statistical test(s) used AND whether they are one- or two-sided<br>*Only common tests should be described solely by name; describe more complex techniques in the Methods section.*
☒	☐	A description of all covariates tested
☒	☐	A description of any assumptions or corrections, such as tests of normality and adjustment for multiple comparisons
☒	☐	A full description of the statistical parameters including central tendency (e.g. means) or other basic estimates (e.g. regression coefficient) AND variation (e.g. standard deviation) or associated estimates of uncertainty (e.g. confidence intervals)
☒	☐	For null hypothesis testing, the test statistic (e.g. *F*, *t*, *r*) with confidence intervals, effect sizes, degrees of freedom and *P* value noted<br>*Give P values as exact values whenever suitable.*
☒	☐	For Bayesian analysis, information on the choice of priors and Markov chain Monte Carlo settings
☒	☐	For hierarchical and complex designs, identification of the appropriate level for tests and full reporting of outcomes
☒	☐	Estimates of effect sizes (e.g. Cohen's *d*, Pearson's *r*), indicating how they were calculated

Our web collection on statistics for biologists contains articles on many of the points above.

Software and code

Policy information about availability of computer code

Data collection	All developed code and models are made publicly available on our AI-CVI laboratory's GitHub page (https://github.com/AI-in-Cardiovascular-Medicine/AAOCA) under the Attribution-NonCommercial 4.0 International (CC BY-NC 4.0) licenses. Permanent reference to the version of the code (V1.0.0) used in this study provided in Zenodo (https://doi.org/10.5281/zenodo.14870126).<br>In addition, we have also provided a publicly available web service, accessible via the following link (Link to the project: https://mb-neuro.medical-blocks.ch/public_access/projects and link to the WebApp: https://mb-neuro.medical-blocks.ch/public_access/projects/aaoca), which allows users to easily upload images in various desired formats for use to get the report and result based on models developed in the current study.
Data analysis	Please see the " https://github.com/AI-in-Cardiovascular-Medicine/AAOCA"for more detailed information.

For manuscripts utilizing custom algorithms or software that are central to the research but not yet described in published literature, software must be made available to editors and reviewers. We strongly encourage code deposition in a community repository (e.g. GitHub). See the Nature Portfolio guidelines for submitting code & software for further information.

Data

Policy information about availability of data

All manuscripts must include a data availability statement. This statement should provide the following information, where applicable:

- Accession codes, unique identifiers, or web links for publicly available datasets
- A description of any restrictions on data availability
- For clinical datasets or third party data, please ensure that the statement adheres to our policy

The dataset used for segmentation model development and clinical evaluation is publicly available (Link: https://zenodo.org/records/6802614). The fully anonymized datasets from Bern and Zurich allow restricted access only, in accordance with the requirements of the institutional review board (IRB) approvals and data sharing regulations. The raw datasets from Bern and Zurich University are protected and are not available due to data privacy laws. Access can be obtained upon IRB and Data Sharing Committee approvals from Bern, Zurich, and the requesting institution, within a time frame of one year. Details on how to request access are available from Dr. Christoph Gräni. The dataset from Guangdong Provincial People's Hospital (external clinical evaluation dataset), which can be used to test and evaluate different segmentation and classification models, is publicly available in(Link: https://www.kaggle.com/datasets/xiaoweixumedicalai/imagecas). Source data are provided with this paper. Corresponding code for the source data, to regenerate the figures and tables are also provided with this paper.

Research involving human participants, their data, or biological material

Policy information about studies with human participants or human data. See also policy information about sex, gender (identity/presentation), and sexual orientation and race, ethnicity and racism.

Reporting on sex and gender	We did not apply any selection criteria related to sex or gender. Biological sex, based on patient self-reporting, was gathered from registries and CT DICOM image headers and was considered in the study design to ensure the generalizability of our findings. All analyses were disaggregated based on patient sex and reported in the supplemental datasets.
Reporting on race, ethnicity, or other socially relevant groupings	In our study, we do not have any socially constructed or socially relevant categorization variables.
Population characteristics	We provided gender, age, and BMI as covariates to characterize the relevant population for our human research participants. In the train dataset from Bern (No. 536), the median age was 61.0 years [Q1: 54.0, Q3: 67.0], with 37.3% females and a median BMI of 25.5 [23.0, 28.7]. The internal test dataset from Ben (No. 359) had a median age of 62.0 years [55.0, 69.0], 35.1% females, and a median BMI of 25.7 [23.4, 29.1]. In the external test dataset from Zurich (No. 483), the median age was 57.0 years [50.0, 64.0], with 34.2% females and a median BMI of 25.9 [23.2, 28.7].
Recruitment	More detailed information about the datasets can be accessed through ClinicalTrials.gov: the Registry for Invasive and Non-invasive Anatomical Assessment and Outcome of Coronary Artery Anomalies (NARCO: NCT04475289) and Clinical Utility and Outcome Prediction of Cardiovascular Computed Tomography (PREDICT-CT: NCT04827316). Both datasets include all patients aged ≥18 years who provided informed consent and underwent CCTA, with no exclusion criteria. For the NARCO dataset, additional data were collected from patients with coronary artery anomalies (CAA) undergoing clinically indicated testing (non-invasive and/or invasive) at our institution.
Ethics oversight	All procedures in studies involving human participants adhered to the ethical standards of the institutional and/or national research committee, the 1964 Helsinki Declaration, and its subsequent amendments or comparable ethical standards. The Bern (KEK 2020-00841 and KEK 2021-0058) and Zurich (KEK 2015-0235 and KEK 2014-0632) cantonal ethics committee approved the study design for the dataset used in the current study. Participants in the study provided written informed consent prior to any data collection, and all imaging data were anonymized.

Note that full information on the approval of the study protocol must also be provided in the manuscript.

Field-specific reporting

Please select the one below that is the best fit for your research. If you are not sure, read the appropriate sections before making your selection.

☒ Life sciences ☐ Behavioural & social sciences ☐ Ecological, evolutionary & environmental sciences

For a reference copy of the document with all sections, see nature.com/documents/nr-reporting-summary-flat.pdf

Life sciences study design

All studies must disclose on these points even when the disclosure is negative.

Sample size	No formal sample size calculation was performed, as there are currently no established methods for determining sample sizes in AI studies. AI models generally benefit from the largest dataset possible, which was also evident in our study—where increasing the training dataset size led to a reduction in false positive cases. Therefore, we included all available data, excluding only cases that met the predefined exclusion criteria outlined in the manuscript (mentioned in the next section). As the model successfully converged during training and testing and performed well across different datasets, the dataset size for this task is sufficient.

Data exclusions	We excluded patients without contrast-enhanced cardiac CT images, those with uninterpretable images due to severe artifacts, images with cropped regions of the aorta, and those without a report for the origin and anatomical risk classification were excluded from the analysis. Overall, we excluded from the analysis 14 patients without contrast-enhanced cardiac CT images, 10 with uninterpretable images due to severe artifacts, 6 patients with images with cropped regions of the aorta, and 12 without a report for the origin and anatomical risk classification.
Replication	We reported all results using an untouched internal test set, as well as an external test set obtained from another institution and city in Switzerland. Additionally, we validated the model on a completely different population from China, representing a distinct geography and demographic. Across all test sets, the model demonstrated robust performance, with reproducible results. Furthermore, we have made all code and models publicly available, ensuring that the entire research process can be independently reproduced. Moreover we repeated the process multiple time and all results were reproducible.
Randomization	In the Bern dataset, randomization was performed based on the definitions of retrospective and prospective cohorts in NARCO and PREDIC-CT data. Datasets from other locations, including Zurich and China, were used as external datasets, representing separate groups.
Blinding	Blinding is not applicable to this study due to the distinct definitions and separation of data cohorts.

Reporting for specific materials, systems and methods

We require information from authors about some types of materials, experimental systems and methods used in many studies. Here, indicate whether each material, system or method listed is relevant to your study. If you are not sure if a list item applies to your research, read the appropriate section before selecting a response.

Materials & experimental systems

n/a	Involved in the study
☒	☐ Antibodies
☒	☐ Eukaryotic cell lines
☒	☐ Palaeontology and archaeology
☒	☐ Animals and other organisms
☐	☒ Clinical data
☒	☐ Dual use research of concern
☒	☐ Plants

Methods

n/a	Involved in the study
☒	☐ ChIP-seq
☒	☐ Flow cytometry
☒	☐ MRI-based neuroimaging

Clinical data

Policy information about clinical studies

All manuscripts should comply with the ICMJE guidelines for publication of clinical research and a completed CONSORT checklist must be included with all submissions.

Clinical trial registration	ClinicalTrials.gov:<br>Registry for Invasive and Non-invasive Anatomical Assessment and Outcome of Coronary Artery Anomalies (NARCO: NCT04475289)<br>Clinical Utility and Outcome Prediction of Cardiovascular Computed Tomography (PREDICT-CT: NCT04827316)
Study protocol	ClinicalTrials.gov:<br>NARCO: NCT04475289<br>PREDICT-CT: NCT04827316
Data collection	More detailed information about the datasets can be accessed through ClinicalTrials.gov: the Registry for Invasive and Non-invasive Anatomical Assessment and Outcome of Coronary Artery Anomalies (NARCO: NCT04475289) and Clinical Utility and Outcome Prediction of Cardiovascular Computed Tomography (PREDICT-CT: NCT04827316). Both datasets include all patients aged ≥18 years who provided informed consent and underwent CCTA, with no exclusion criteria. For the NARCO dataset, additional data were collected from patients with coronary artery anomalies (CAA) undergoing clinically indicated testing (non-invasive and/or invasive) at our institution. The dataset used for segmentation model development and clinical evaluation is publicly available(Link: https://zenodo.org/records/6802614). The datasets from Bern (2009-2024) and Zurich (2021-2023) University Hospitals (training, internal validation, internal and external testing set) are not shareable due to a lack of dedicated ethical approval for this purpose. The dataset from Guangdong (2012-2018) Provincial People's Hospital (external clinical evaluation dataset), which can be used to test and evaluate different segmentation and classification models, is publicly available in (Link: https://www.kaggle.com/datasets/xiaoweixumedicalai/imagecas).
Outcomes	Our study does not focus on outcomes but rather on diagnoses made by cardiologists specialized in CCTA image analysis.

Plants

Seed stocks	n/a
Novel plant genotypes	n/a
Authentication	n/a
